# Mechanisms of NLRP3 inflammasome in chronic kidney disease and the effects of traditional Chinese medicines

**DOI:** 10.1080/0886022X.2025.2538798

**Published:** 2025-08-21

**Authors:** Yushan Sun, Guanghui Pan, Chunxing Wang, Zhenwei Xiao

**Affiliations:** ^a^ First College of Clinical Medicine, Shandong University of Traditional Chinese Medicine, Jinan, China; ^b^ Affiliated Hospital of Shandong, University of Traditional Chinese Medicine, Jinan, China; ^c^ Department of First Clinical Medical College, Shandong University of Traditional Chinese Medicine, Jinan, China

**Keywords:** Chronic kidney disease, NLRP3 inflammasome, mechanism, plant metabolites, traditional Chinese medicine

## Abstract

Chronic kidney disease (CKD) is characterized by a progressive decline of renal function, leading to multisystem problems and finally culminating in end-stage renal disease (ESRD). It has progressively emerged as a major global health issue. The nucleotide-binding oligomerization domain-like receptor pyrin domain-containing 3 (NLRP3) inflammasome contributes to the inflammatory response chiefly by facilitating the secretion of inflammatory mediators, including interleukin-1β and interleukin-18. Recent studies have underscored the significance of NLRP3 inflammasome in renal pathology that is associated with CKD. In addition, a large number of studies have shown that traditional Chinese medicines (TCMS) can regulate NLRP3 through multiple targets and pathways, demonstrating significant potential in the treatment of CKD. These natural products offer advantages such as low costs and minimal side effects, making them a viable supplement or alternative to traditional treatment approaches for CKD. A literature review approach was employed to systematically search databases including Web of Science, ScienceDirect, and PubMed. Search keywords comprised ‘Chronic kidney disease’, ‘NLRP3 inflammasome’, ‘traditional Chinese medicine’, and their relevant synonyms/combinations. Through reviewing abstracts and full texts, the relationships between CKD and NLRP3 inflammasome, as well as the pharmacological mechanisms of TCMS, were classified and summarized. This study also explores the therapeutic potential of TCMS in regulating NLRP3 inflammasome. These findings might offer prospective diagnostic and therapeutic approaches for targeted or supplementary therapy of CKD.

## 1. Introduction

Chronic kidney disease (CKD) refers to a long-term, irreversible decline in renal function, characterized by the progressive loss of kidney function that can ultimately lead to renal failure [1]. CKD has emerged as a critical global health challenge, ranking among the leading threats alongside cardiovascular diseases, diabetes, and cancer, due to its high prevalence, low awareness, complex complications, poor prognosis, and substantial medical costs. The global median prevalence of CKD is 9.5%, and with the aging population and rising incidence of conditions like diabetes and hypertension, its prevalence continues to increase [2–4].

Current management of CKD primarily comprises three stages: early drug therapy, dialysis, and kidney transplantation. Early pharmacotherapy plays a pivotal role in delaying CKD progression, including interventions with renin-angiotensin system blockers and sodium-glucose cotransporter 2 inhibitors. Treatment also involves managing complications such as anemia and electrolyte disorders, as well as nutritional support through essential amino acid supplementation to maintain overall patient health [3]. However, despite the efficacy of existing therapies in symptom management, the high morbidity and mortality associated with CKD remain a significant concern, making precise targeted treatment a focal point of current research.

The nucleotide-binding oligomerization domain-like receptor pyrin domain-containin g 3 (NLRP3) inflammasome, a cytoplasmic cysteine-aspartic acid protease-activating complex first proposed by Martinon in 2002, participates in innate immune responses [5]. As the most widely studied inflammation to date, it responds to diverse stimuli and plays a central role in the pathogenesis of numerous inflammatory diseases. Research has highlighted the critical role of the NLRP3 inflammasome in multi-organ inflammatory disorders, making the study of its regulatory mechanisms essential for developing novel anti-inflammatory therapies. In the liver, it is associated with nonalcoholic steatohepatitis and viral hepatitis, where overactivation exacerbates inflammatory responses and hepatocyte damage, driving disease progression. In the heart, it is linked to atherosclerosis, myocardial infarction, and heart failure, promoting atherosclerotic plaque formation, increasing cardiovascular event risk, and worsening myocardial cell damage post-infarction, thereby impacting cardiac function recovery. In the brain, abnormal activation is associated with neurodegenerative diseases such as Alzheimer’s and Parkinson’s, inducing neuroinflammation and cell death that accelerate cognitive and motor decline [6–8].

In recent years, scientific evidence has established a close association between CKD and the NLRP3 inflammasome. Excessive NLRP3 inflammasome activation is considered a key factor contributing to renal tissue damage and dysfunction, playing a significant role in inducing renal inflammation and fibrosis [9]. Renal tissue from CKD patients, particularly in renal tubular epithelial cells (RTEC) and macrophages, exhibits a notable upregulation of NLRP3 inflammasome expression [10]. This expression increase is not only closely linked to local renal inflammatory responses but may also exacerbate kidney damage through complex mechanisms, including ion flux alterations, organelle damage, ferroptosis, autophagy dysregulation, and modulation of intestinal flora metabolism.

Currently, small-molecule inhibitors targeting NLRP3 inflammasome components are being explored as potential therapeutics for kidney-related diseases [11]. Moreover, numerous studies have indicated that traditional Chinese medicines (TCMS) can effectively modulate NLRP3 inflammasome activity through its multi-component, multi-pathway properties, thereby improving symptoms and prognosis in CKD patients [12–14]. This review aims to summarize the structure and function of the NLRP3 inflammasome, discuss its relationship with CKD, and outline how TCMS delays CKD progression by intervening in NLRP3 inflammasome activation, seeking to provide a comprehensive theoretical foundation and clinical perspective for CKD prevention and treatment.

## 2. Overview of the NLRP3 inflammasome

The NLRP3 inflammasome primarily consists of three key proteins: the apoptosis-associated speck-like protein containing a CARD (ASC), the NLRP3 protein, and pro-cysteine aspartic acid-specific protease-1 (Pro-caspase-1) [15,16]. The CARD of ASC enables the recruitment of Pro-caspase-1. As an adaptor protein, ASC possesses two domains—the pyrin domain and caspase recruitment domain—which act as a ‘bridge’ to connect receptor and effector proteins, thereby forming an efficient signal transduction complex [17]. NLRP3 serves as the core component of the inflammation, comprising three major domains: the N-terminal pyrin domain (PYD), the C-terminal leucine-rich repeat (LRR) domain, and the centrally located NACHT domain (also referred to as the nucleotide-binding oligomerization domain). The PYD interacts with ASC *via* PYD-PYD binding, while the nucleotide-binding oligomerization domain primarily promotes self-oligomerization. The LRR domain is predominantly responsible for stimulus recognition [18].

## 3. Activation pathway of the NLRP3 inflammasome

The NLRP3 inflammasome activates in response to diverse pathogen-associated molecular patterns (PAMPs) and damage-associated molecular patterns (DAMPs). Its primary function is to activate caspase-1, which in turn induces the maturation and secretion of pro-inflammatory cytokines interleukin-1β (IL-1β) and interleukin-18 (IL-18). Caspase-1 also cleaves the membrane perforin gasdermin D (GSDMD), leading to cell lysis and release of inflammatory mediators [16,19]. NLRP3 inflammasome activation occurs in two distinct phases: ‘priming’ and ‘activation’ [6,20–22].

The priming phase is initiated by pattern recognition receptor-mediated signaling, including tumor necrosis factor-α (TNF-α) and toll-like receptor (TLR4) signaling. This signaling activates inflammatory vesicle components such as NLRP3, IL-1β, and IL-18 through modulation of the nuclear factor-kappa B (NF-κB) signaling pathway and other transcriptional processes [22–24]. Activation signals are triggered by PAMPs and DAMPs, including pore-forming toxins, ATP, RNA viruses, urate crystals, ion flux disturbances, reactive oxygen species (ROS) production, mitochondrial dysfunction, and lysosomal damage [23,25]. During the activation of NLRP3 inflammasome, the NACHT domain of NLRP3 binds to LRRs, maintaining a state of self-inhibition. When PAMPs and DAMPs appear, NLRP3 releases its self-inhibition state, exposes the NACHT domain, and oligomerizes. NLRP3 then undergoes auto-oligomerization to form an open octameric structure. Interactions between NACHT domains of NLRP3 initiate oligomerization, after which its PYD interacts with the PYD of ASC, promoting ASC oligomerization. The oligomerized ASC further assembles into helical filamentous structures and eventually forms large aggregates called ‘ASC specks’ [26]. Under normal conditions, NLRP3 and ASC are diffusely distributed in the cytoplasm. Upon NLRP3 activation, both proteins rapidly migrate and form punctate structures around the nucleus—the ASC specks [27]. Knockdown of endogenous ASC expression or inhibition of ASC oligomerization significantly suppresses NLRP3. Pro-casepase-1 is recruited into the inflammation complex through the interaction between its CARD and the CARD of ASC, subsequently undergoing autocatalysis to cleave itself into active caspase-1. Once the components of the inflammation assemble into an active complex, caspase-1 is activated. This activation initiates the secretion of downstream mature pro-inflammatory cytokines, such as IL-1β and IL-18 [28]. Simultaneously, activated caspase-1 can also cleave the key pro-inflammatory protein GSDMD to C-GSDMD and N-GSDMD [29,30]. After N-GADMD is activated and released, pores are formed on the membrane through cell membrane lipid interactions, resulting in the release of IL-1β and IL-18 to the outside of the cell and inducing pyroptosis [19]. IL-1β and IL-18 can not only recruit and activate immune cells to aggravate the inflammatory response, but also may affect RTEC through autocrine or paracrine pathways, aggravating the pathological process of renal tubular injury and fibrosis by influencing their proliferation and apoptosis [31].

However, beyond the classical pathways, there are non-classical activation pathways for the NLRP3 inflammasome. When dangerous signals such as lipopolysaccharide (LPS) are detected in the cytoplasm, caspase-11 (in mice) or caspase-4/5 (in humans) are activated, which is considered to be the starting point of non-classical pathways [32,33]. Recent studies have found that Nur77 can function as an intracellular LPS sensor, binding to mitochondrial DNA and LPS to activate the non-classical NLRP3 inflammasome pathway, thereby regulating the body’s immune response [34,35].

**Figure 1.**
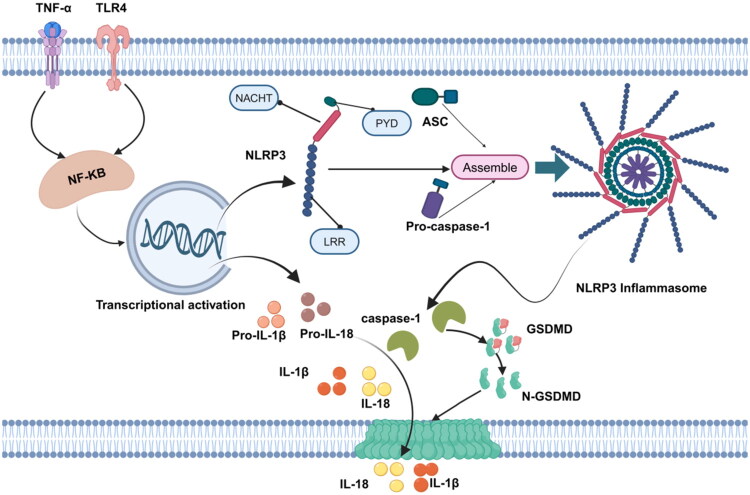
Classical signaling pathway of the NLRP3 inflammasome [20]. TNF-α: Tumor Necrosis Factor-α;TLR4: Toll-like Receptor 4; NF-κB: Nuclear Factor-Kappa B; NACHT: NAIP, CIITA, HET-E, TP1 domain; PYD: Pyrin Domain; ASC: Apoptosis-associated Speck-like Protein containing a CARD; NLRP3: Nucleotide-binding Oligomerization Domain-Like Receptor Family Pyrin Domain Containing 3; LRR: Leucine-rich Repeat; Pro-caspase-1: Pro-cysteine aspartic acid protease-1; Pro-IL-1β: Pro-Interleukin-1β; Pro-IL-18: Pro-Interleukin-18; IL-1β: Interleukin-1β; IL-18: Interleukin-18; GSDMD: Gasdermin D; N-GSDMD: N-terminal fragment of Gasdermin D. (Created in https://BioRender.com)

Additionally, while NLRP3 typically functions as part of the inflammation complex, it also possesses some independent functions. More and more studies have revealed that the NLRP3 protein can participate in the pathogenesis of diseases independently of the inflammation NLRP3-ASC-caspase-1-IL-1β-IL-18 signal axis. NLRP3 can also form a complex with ASC and caspase-8 in mitochondria, thereby regulating the apoptosis of renal and intestinal epithelial cells [36,37]. Other research has shown that the Z-DNA binding protein 1-NLRP3 inflammasome can be specifically activated by viral RNA products or endogenous nucleic acid ligands, which have been shown to promote a mixed form of cell death-pan-apoptosis (Figure 1) [38].

## 4. Mechanisms of NLRP3 inflammasome

**Figure 2.**
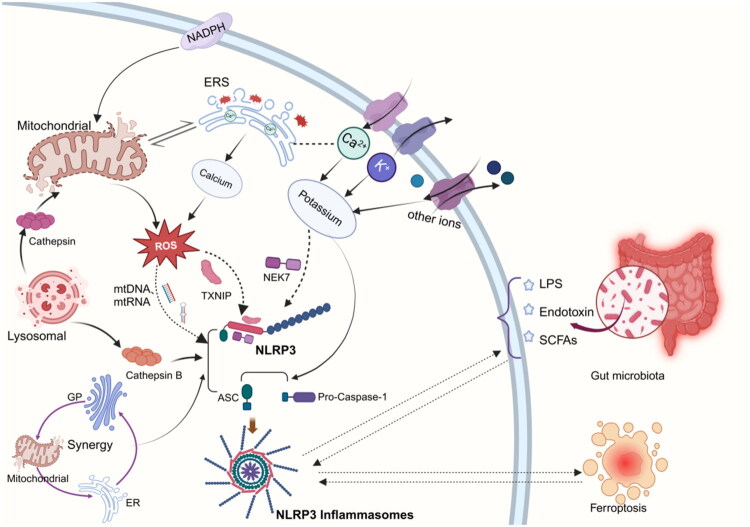
Mechanisms of the NLRP3 inflammasome activation [39,40]. NADPH: Nicotinamide Adenine Dinucleotide Phosphate; ERS: Endoplasmic Reticulum Stress; ROS: ROS; mtDNA: Mitochondrial DNA; mtRNA: Mitochondrial RNA; TXNIP: Thioredoxin-interacting Protein; NEK7: NIMA-related Kinase 7; LPS: Lipopolysaccharide; SCFAs: Short-Chain Fatty Acids; ASC: Apoptosis-associated Speck-like Protein containing a CARD; Pro-Caspase-1: Pro-cysteine-aspartic acid Protease-1; NLRP3: Nucleotide-binding Oligomerization Domain-Like Receptor Family Pyrin Domain Containing 3. (Created in https://BioRender.com)

Activation of the NLRP3 inflammasome entails multiple molecular and cellular events, along with dysregulations in internal environmental homeostasis. These include ion flux alterations, organelle damage, pyroptosis, ferroptosis, autophagy, and metabolic modulation of the intestinal microbiota [39–44]. Additionally, while NLRP3 proteins are involved in assembling NLRP3 inflammasomes to execute their functions, they can also influence the pathophysiology of CKD independently of inflammation complexes (Figure 2).

### 4.1. Ion flux

#### 4.1.1. Potassium ions

Potassium ion (K^+^) efflux is recognized as a key upstream event in NLRP3 inflammasome activation [45,46]. In pathological states (e.g., infection, tissue injury), increased extracellular K^+^ concentrations act as danger signals, triggering activation of the NLRP3 inflammatory complex. Notably, NLRP3 inflammasome activation can occur even in the absence of activators by removing K^+^ from cell culture media, highlighting the potential significance of K^+^ in NLRP3 inflammasome signaling.

The role of NLRP3 as a sensor for intracellular K^+^ efflux has been confirmed. NIMA-associated kinase 7 (*NEK7*), a protein that binds to NLRP3, has emerged as a critical player in the K^+^ efflux downstream pathway. During K^+^ efflux, *NEK7* interacts with the LRR domain of NLRP3, promoting its structural rearrangement and oligomerization, which in turn triggers inflammation activation [47–49]. Most studies have focused on the role of NLRP3 protein and K^+^ in inflammation activation. However, there is currently no direct evidence to show that K^+^ can interact directly with the NLRP3 protein. Another study has shown that a reduction in intracellular K^+^ levels not only triggers the NLRP3 response but also induces structural changes in ASC oligomers and enhances the recruitment of the Pro-caspase-1 to ASC specks, a process that is independent of NLRP3 [50]. This implies that the role of K^+^ in the activation of inflammations may be multidimensional. In addition, the activation of NLRP3 inflammasome triggered by certain small molecule compounds, such as imiquimod and chlorfenuron, seems to be independent of K^+^ outflow but relies on the production of mitochondrial reactive oxygen species (mtROS), indicating that K^+^ outflow may not be an essential prerequisite for the activation of NLRP3 inflammasome [51,52].

Despite a lack of direct studies to definitively clarify the direct interaction of potassium ions with the NLRP3 inflammasome in CKD, it is possible that K^+^ may be indirectly implicated in the inflammatory response and pathological process of CKD by influencing NLRP3 activation.

#### 4.1.2. Calcium ions

Elevated cytosolic calcium (Ca^2+^) is frequently required for NLRP3 inflammasome activation. Various NLRP3 inflammasome agonists, including cytoplasmic Ca^2+^ influx and ROS, can activate intracellular Ca^2+^ signaling cascades *via* calcium receptor (CASR) interaction with phospholipase C, consequently influencing NLRP3 [53–55].

Researchers have found that lipopolysaccharide and palmitic acid decrease lysosomal Ca^2+^ levels while increasing cytoplasmic Ca^2+^ *via* ROS, triggering NLRP3 inflammasome activation. Blocking Ca^2+^ channels in the cell membrane can prevent caspase-1 activation and IL-1β production [56], suggesting that Ca^2+^ may act on inflammation downstream components. Pharmacological studies showed that inhibiting large-conductance calcium-activated potassium channels suppress silica-induced lysosomal membrane damage and NLRP3 inflammasome activation [57]. However, the mechanism by which intracellular Ca^2+^ elevation promotes NLRP3 activation remains unclear. Notably, K^+^ efflux typically occurs concurrently with Ca^2+^ influx during NLRP3 inflammasome activation [58]. A cell experiment demonstrated that when directly triggering rapid cytoplasmic K^+^ efflux, extracellular Ca^2+^ influx and cytoplasmic Ca^2+^ elevation are not essential signals for NLRP3 inflammasome activation [59]. Thus, Ca^2+^ flux may serve as a regulator—rather than an absolute requirement—for NLRP3 inflammasome activation.

#### 4.1.3. Other ions

As existing research deepens, increasing evidence indicates that ion fluxes such as Na^+^, Cl^−^, Fe^2+^, Zn^2+^, and Mn^2+^ also play a significant role in the assembly of the NLRP3 inflammasome [39,60–63]. However, currently, these ions appear to act synergistically with K^+^ and cannot independently activate the NLRP3 inflammasome. In the future, more studies are needed on the potential ionic mechanisms.

### 4.2. Organelle damage

#### 4.2.1. Mitochondrial damage

Due to the kidney’s unique high oxygen consumption characteristics, the mitochondria in its cells are not only abundant but also exhibit high adaptability and repair capabilities. However, persistent mitochondrial damage and excessive activation of inflammations can induce inflammatory responses and tissue damage [64,65]. There is a potential interaction mechanism between mitochondrial damage and the activation of the NLRP3 inflammasome [66,67]. In transforming growth factor-β-treated (TGF-β) RTEC and animal models of renal injury, researchers have found that peroxisome proliferator - activated receptor - gamma coactivator 1-α (PGC-1α) can inhibit the activation of the NLRP3 inflammasome pathway by reducing mitochondrial damage and restoring mitochondrial integrity, thereby reducing renal cell damage and fibrosis [68]. This reveals the importance of regulating mitochondrial activity and dynamics in modulating the NLRP3 inflammasome signaling pathway.

Damage to mitochondria results in dysfunction of the mitochondrial electron transport chain, leading to a significant increase in the generation of mtROS [69]. This process promotes the oligomerization of NLRP3—a crucial factor in the activation of the NLRP3 inflammasome. Thioredoxin-interacting protein (TXNIP) is an essential element of the thioredoxin system. It can significantly influence the body’s reaction to oxidative stress [70–73]. The overproduction of mtROS can impede the antioxidant activity of the endogenous antioxidant thioredoxin, leading to its dissociation from TXNIP. The separated TXNIP binds to the NLRP3 inflammasome and promotes the activation of the NLRP3 inflammasome, thereby exacerbating renal tubular injury and fibrosis. Knockout of the NLRP3 gene markedly diminishes inflammation activity indicators, improving kidney injury [40,74–76].

Furthermore, nicotinamide adenine dinucleotide phosphate (NADPH) oxidase, a key enzyme in the production of ROS, has been demonstrated to be closely associated with the activation of the NLRP3 inflammasome. NADPH oxidase can result in the overproduction of ROS [77–80]. In a mouse model of CKD, inhibiting the principal pathway of NADPH oxidase/ROS/NLRP3 inflammasome effectively diminishes the inflammatory response in the kidneys and reduces the extent of fibrosis [81]. The activation of mtROS also exacerbates the release and synthesis of oxidized mitochondrial DNA, mitochondrial RNA, and other mitochondrial-related proteins and lipids, which in turn further activate the NLRP3 inflammasome as DAMPs [82,83]. However, in contrast to the aforementioned perspective, recent innovative research by Billingham and his team has discovered that macrophages can still activate NLRP3 signaling in response to various NLRP3 agonists, even in the absence of mtROS [69]. This suggests that mtROS may not be essential for the activation of NLRP3.

#### 4.2.2. Lysosomal damage

The lysosome serves as the core degradation center within cells. Damage to lysosomes is frequently associated with various kidney diseases [84,85], and the NLRP3 inflammasome also plays a crucial role in this pathological process.

Alterations in lysosomal protease activity have been observed during renal pathology. Research indicates that lysosomal acidification-mediated cathepsin, such as cathepsin B, L, C, S, and X, are implicated in the activation of the NLRP3 inflammasome, particularly the release of lysosomal cathepsin B [86–89]. Mature cathepsin B facilitates the interaction between NLRP3 and ASC, and promotes ASC oligomerization to form plaques, which is crucial for NLRP3 activation. Zheng et al. demonstrated through *in vitro* and *in vivo* experiments that the activities of lysosomal cathepsins B, S, and L are increased in cells with activated NLRP3 inflammasomes. Treatment with flufenidone significantly downregulates cathepsin activity. Under inflammatory conditions induced by LPS/ATP stimulation or hypoxia/reoxygenation treatment, cathepsin B is released from lysosomes and co-localizes with NLRP3 in the cytoplasm. Flufenidone reduces NLRP3 inflammasome activation by inhibiting cathepsin B release [90]. Concurrently, leaked lysosomal cathepsin can directly impact mitochondria, causing mitochondrial damage and ROS production [91], and can induce NLRP3 inflammasome activation *via* the ROS/TXNIP/NLRP3 signaling pathway, suggesting that lysosomal damage may activate inflammations by disrupting mitochondrial function [73]. Further research has indicated that this process might be mediated by affecting the flow of calcium ions within lysosomes. Apilimod is a compound that disrupts normal lysosomal function. By compromising lysosomal integrity and altering the acidic environment within lysosomes, Apilimod triggers lysosome-associated signaling pathways to activate the NLRP3 inflammasome. Specifically, Apilimod activates the transient receptor potential mucolipin 1 calcium channel on lysosomes, prompting calcium release from lysosomes into the cytoplasm. Calcium overload subsequently disrupts mitochondrial membrane potential, increasing mtROS production, mtROS then oxidatively modify components of the NLRP3 inflammasome, ultimately leading to its activation [92]. On the contrary, calcium chelators and lysosomal calcium channel inhibitors can eliminate Apilimod-induced mitochondrial damage and NLRP3 inflammasome activation [92].

#### 4.2.3. Endoplasmic reticulum stress

The endoplasmic reticulum (ER) is a critical organelle tasked with protein synthesis, folding, and modification within cells. The buildup of unfolded or misfolded proteins in the ER can result in endoplasmic reticulum stress [93]. Multiple pathogenic causes, such as free fatty acids, angiotensin II, advanced glycation end products, and hyperglycemia, can trigger endoplasmic reticulum stress (ERS). These variables disturb the balance of the ER and ultimately result in the aggregation of misfolded proteins. The unfolded protein responses subsequently trigger the activation of the NLRP3 inflammasome [41,94].

During ERS, ROS not only act as signaling molecules to modulate mitochondrial function and cellular redox status but also promote the interaction and activation of NLRP3 inflammasome components (e.g., NLRP3, ASC, and caspase-1) through oxidative modifications. During ERS, the release of Ca^2+^ from the ER is a crucial signaling event. After entering the cytosol, Ca^2+^ activates calcineurin, which in turn regulates the activity of transcription factors including NF-κB. Once activated, NF-κB directly binds to the promoter region of the NLRP3 gene, facilitating its transcription and thereby promoting the expression of NLRP3 inflammasome-related genes. Importantly, ERS also promotes physical contact between the ER and mitochondria through dysregulation of calcium signaling [41,95].

#### 4.2.4. Other organelles

As a central organelle for protein processing, modification, and transport, the Golgi apparatus has emerged as a key player in inflammation activation. Recent studies show that golgi-targeted photodynamic therapy significantly up-regulates NLRP3 expression and promotes release of pro-inflammatory cytokines (e.g., IL-1β, IL-18), underscoring its critical role in NLRP3 inflammasome activation. While the regulatory mechanisms between the golgi and NLRP3 remain partially elucidated, cross-organelle crosstalk—including interactions with the ER and mitochondria—is recognized as central to NLRP3 inflammasome priming and assembly. Zhang et al. demonstrated that upon NLRP3 inflammasome activation, mitochondria-associated membranes localize adjacent to the Golgi, coinciding with increased Golgi diacylglycerol levels [96]. This triggers recruitment and activation of protein kinase D, which phosphorylates NLRP3. Phosphorylated NLRP3 dissociates from mitochondria-associated membranes and undergoes ordered cytoplasmic assembly into functional inflammations. This pathway confirms that Golgi-mediated protein kinase D signaling is indispensable for NLRP3 inflammasome activation [97].

Notably, current research on NLRP3 inflammasomes in CKD has primarily focused on mitochondria, ER, and lysosomes, or their collaborative interactions.

### 4.3. Cell pyroptosis and pan-apoptosis

Pyroptosis is a type of programmed cell death that depends on inflammatory caspases, chiefly caspase-1, 4, 5, and It is marked by cell swelling and the secretion of pro-inflammatory cytokines. The activation of the NLRP3 inflammasome can induce pyroptosis in renal cells, whereas its inhibition can decelerate this process, hence mitigating the advancement of CKD [98–100]. Li et al. found that Bushen Huoxue Granule can effectively block the activation of the NLRP3 inflammasome triggered by angiotensin II in human proximal tubular epithelial cells (HK-2 cells), thus reducing renal fibrosis and pyroptosis [101].

PANoptosis is a newly discovered form of inflammatory programmed cell death that involves the interactions and communications among pyroptosis, apoptosis, and necroptosis [102]. This procedure has garnered increased attention in recent years. Among them, necroptosis represents a distinct form of programmed necrotic cell death characterized by prominent cellular swelling, plasma membrane rupture, and the subsequent release of intracellular contents. Notably, DAMPs such as ATP, released by necroptotic cells, play a pivotal role in activating the NLRP3 inflammasome. Specifically, ATP mediates K^+^ efflux through the P2X 7 receptor, a critical upstream event that initiates NLRP3 inflammasome assembly and subsequent pyroptosis [103]. PANoptosis may affect the expression of inflammatory factors in RTEC. The pan-apoptotic pathway may be closely associated with the NLRP3 inflammasome. In the TAK1 knockout mouse model, the inhibition or deletion of TAK1 results in pan-apoptosis mediated by the receptor-interacting protein kinase 1-PANoptosome complex. This complex includes the NLRP3 inflammasome along with critical components such as caspase-8, fas-associated death domain, and receptor-interacting protein kinase 3 [43]. More *in vivo* and *in vitro* experiments are needed in the future to fully understand how pan-apoptosis and the NLRP3 inflammasome work in nephrology.

### 4.4. Ferroptosis

Ferroptosis is a form of programmed cell death first proposed in 2012 [104]. It is a new iron-dependent non-apoptotic cell death pathway and is distinct from traditional cell death modes such as apoptosis, pyroptosis, and necrosis. Ferroptosis is characterized by the excessive accumulation of lipid peroxides and ROS. Recent research has shown that reducing ferroptosis in RTEC can reduce the occurrence of renal fibrosis, therefore contributing to the deceleration of CKD progression [105–107].

A complicated relationship may exist between ferroptosis and NLRP3. Ferroptosis can initiate the activation of the NLRP3 inflammasome [108], which subsequently intensifies ferroptosis [109]. The interplay between ferroptosis and NLRP3 in CKD is significant. CD1c^+^ dendritic cells (CD1c^+^ DCs), a subset of myeloid dendritic cells (mDCs), are capable of sensing pathogen-associated and damage-derived signals to initiate immune responses. They also secrete various cytokines and chemokines such as IL-12, IL-6, and TNF-α, playing a critical role in renal inflammation. Giuliani et al. discovered that ferroptosis transpired in HK-2 cells under hypoxic conditions, and the activation of the NLRP3 inflammasome was noted in CD1c dendritic cells. The researchers also noted that in the vicinity of ferroptotic HK-2 cells, interstitial CD1c^+^ DCs containing ASC speckles exhibit significant accumulation. Treatment with the NLRP3 inhibitor Monash Chemical Compound 950 (MCC950) or genetic knock down of NLRP3 significantly suppresses the maturation and secretion of IL-1β/IL-18 in DCs, while attenuating DC maturation phenotypes and Th1 cell polarization capacity. In the kidneys of mice lacking endothelial-cell-specific Atg7, there was an enhanced buildup of ferritin and upregulation of the NLRP3 inflammasome signaling pathway [110]. Pharmacological reduction of ferroptosis can restore the compromised endothelial barrier and reverse the diminished expression of NLRP3 and IL-1β in the aging murine model, hence mitigating renal tubulointerstitial fibrosis. These findings demonstrate the link between the NLRP3 inflammasome and ferroptosis in CKD. Lipid peroxidation serves as a crucial ‘intermediate stage’ in the induction of ferroptosis [111]; yet, its specific manner of action on the NLRP3 inflammasome remains inadequately clarified within the current understanding of ferroptosis mechanisms. Ferroptosis may concurrently have a synergistic effect with other forms of cell death, including pyroptosis [112].

### 4.5. Intestinal flora metabolism

The gut-kidney axis is a bidirectional signaling network that encompasses the kidney, gut, and its microbiome. The gut-kidney axis idea, derived from ‘enterorenal syndrome,’ was formally created by Meijers in 2011 [113], providing a novel framework for comprehending the interplay between the intestine and the kidney. CKD generally results in dysbiosis of the gut microbiota, leading to elevated concentrations of uremic toxins and intestinal bacteria in the bloodstream. This cascade triggers an inflammatory response characterized by the production of ROS and the release of pro-inflammatory cytokines, thereby accelerating renal fibrosis.

The onset and advancement of CKD may be related to disrupted bidirectional communication between NLRP3 and gut microbiota. An imbalance in intestinal flora may trigger the NLRP3 inflammasome by bacterial toxins in the gut, ultimately leading to inflammatory reactions [114]. Inhibiting the activation of the NLRP3 inflammasome pathway may aid in reestablishing the equilibrium of intestinal microbiota and decelerating the progression of CKD. Recent studies have shown that increased uric acid levels might disturb intestinal microbiota balance and activate the NLRP3 inflammasome, resulting in renal impairment. The knockout of the NLRP3 gene decreased the severity of renal injury, verifying the critical role of NLRP3 activation in this phenomenon [44,115].

Short-chain fatty acids (SCFAs), produced by gut microbiota, have shown the capacity to suppress the production and activation of NLRP3 inflammasomes. In a rat model of diabetic nephropathy, red ginseng berry decreased kidney tissue fibrosis by suppressing NLRP3 inflammasomes. Red ginseng berry enhances intestinal barrier protection and increases beneficial intestinal flora, which is essential for regulating the production of SCFAs [116]. An investigation employing a rat model of CKD revealed that exogenous butyrate supplementation, a short-chain fatty acid, significantly decreased the expression levels of various critical proteins linked to NLRP3 inflammasome-mediated pyroptosis, such as NLRP3, IL-1β, caspase-1, and GSDMD. This intervention significantly reduced renal fibrosis. *In vitro* tests with HK-2 cells showed that adding butyrate greatly decreased the rise in NLRP3-mediated pyroptosis protein levels after TGF-β1 stimulation. This supported the idea that SCFAs can stop NLRP3 inflammasomes from forming [117]. These findings indicate that NLRP3 inflammasome may play a role in the progression of renal fibrosis through mechanisms involving the gut-kidney axis and gut microbiota interactions.

Altering the gut microbiota with therapies such as probiotics and prebiotics may improve the management of kidney disease [118,119]. Probiotics are a category of functional microorganisms based on the theory of microecological balance, which exerts beneficial effects on host health when ingested in sufficient quantities. Specifically, probiotics can stimulate intestinal epithelial cells to secrete mucus and antimicrobial peptides, enhancing the intestinal mucosal barrier function to prevent PAMPs from activating the NLRP3 inflammasome. Additionally, they produce metabolic byproducts such as SCFAs, which inhibit NLRP3 inflammasome activation and regulate intestinal immune homeostasis. Prebiotics, as substrates for probiotics, can be selectively utilized by these microorganisms to promote their proliferation in the intestinal tract. In conclusion, these studies indicate that the NLRP3 inflammasome may affect renal fibrosis *via* the intestine-renal axis and the interaction between intestinal microbiota, presenting additional potential avenues for CKD treatment.

### 4.6. Negative activation mechanisms

In addition to the above activation mechanisms, multiple negative feedback pathways exist within cells to maintain the dynamic balance of the NLRP3 inflammasome, which provides new directions for the treatment of CKD.

Autophagy is considered a negative regulator of the NLRP3 inflammasome. The term ‘autophagy’ was coined by Christian de Duve in 1963. It refers to a cellular degradation process where autophagosomes form to engulf damaged organelles and misfolded proteins, subsequently fuzing with lysosomes to degrade them and maintain cellular homeostasis [120]. Activating autophagy aids in removing damaged organelles and protein aggregates, reduces cell death and inflammation, and regulates the synthesis and degradation of the extracellular matrix, thereby improving renal function and slowing the progression of renal fibrosis [121]. Research reveals that autophagy can restrict the activation of the NLRP3 inflammasome, thus preventing sustained inflammation. The specific mechanisms involve the degradation of NLRP3 inflammasome components by autophagosomes, the clearance of damaged mitochondria to reduce intracellular ROS levels, and the degradation of metabolites like cholesterol crystals and uric acid crystals to prevent their activation of the NLRP3 inflammasome. Atg5, a member of the autophagy-related gene family, forms an essential Atg5-Atg12 complex for autophagosome membrane elongation and closure. Compared to NRK-52E cells, NRK-Atg5-(2) cells with reduced Atg5 expression show significantly upregulated NLRP3, caspase-1, fibronectin, and α-smooth muscle actin. This implies that lowered Atg5 may boost NLRP3 expression, worsening renal inflammation and fibrosis [122]. However, current research on autophagy’s role in regulating the NLRP3 inflammasome to slow CKD progression remains limited. Future studies should explore this link further to identify potential therapeutic targets and strategies for CKD treatment.

Transcriptomic research indicates that microRNAs may negatively regulate NLRP3 activation. These endogenous small non-coding RNAs suppress specific target gene expression post-transcriptionally and play a key role in regulating kidney inflammation, cell injury, fibrosis, and epithelial-mesenchymal transition. In *db/db* and STZ-treated diabetic nephropathy mice, miR-10a/b curbs NLRP3 inflammasome activation, reducing pro-inflammatory cytokines IL-1β and IL-This notably improves renal inflammation and alleviates albuminuria. Conversely, miR-10a/b CKD boosts NLRP3 inflammasome activation, exacerbating renal inflammation [123].

Although negative regulatory mechanisms such as autophagy and microRNAs have been preliminarily revealed, the tissue-specific regulation and intercellular interaction mechanisms of these pathways in the context of CKD still require in-depth study. Targeting the negative regulatory pathways of NLRP3 (such as regulating autophagy or miRNA expression through TCMS may become a new strategy to delay the progression of CKD.

## 5. The role of NLPR3 inflammation in different CKDs

### 5.1. Diabetic nephropathy

Diabetic nephropathy (DN) is the primary cause of ESRD. As an inflammatory disease, DN is largely influenced by secreted inflammatory mediators. The activation of the NLRP3 inflammasome is closely associated with several pathological characteristics of DN, such as glomerular injury, renal interstitial fibrosis, and proteinuria. Inhibiting the activation of the NLRP3 inflammasome can help mitigate the pathological damage caused by DN. Inhibiting the activation of the NLRP3 inflammasome helps alleviate pathological damage in DN [124].

Diabetic metabolic abnormalities, such as hyperglycemia, hyperlipidemia, and elevated free fatty acids, can directly or indirectly trigger the NLRP3 inflammasome. This activation results in the release of IL-1β and IL-18, leading to damage in islet cells, insulin resistance, and systemic inflammation. Studies have demonstrated that elevated glucose levels can upregulate TXNIP expression and activate the NLRP3 inflammasome in renal tissue of DN rats, increasing levels of inflammatory factors in a time- and dose-dependent manner. Silencing TXNIP has been shown to decrease ROS levels and inhibit NLRP3 inflammasome activation [125]. NF-κB p65 is a crucial element of the nuclear factor-kappa B family. It operates as a transcription factor, modulating inflammatory responses. NF-κB p65 is activated downstream of the ROS/NLRP3 signaling pathway and then transferred to the nucleus, which subsequently regulates the transcription of a series of inflammation-related genes. Wu et al. revealed that inhibiting NLRP3 inflammasome activation significantly diminishes NF-κB p65 activation and mtROS generation in podocytes exposed to elevated glucose levels [126]. This emphasizes the essence of the NLRP3/ROS/NF-κB p65 pathway in the accumulation of lipids associated with diabetes in podocytes. In recent years, studies have found that there may be new regulatory mechanisms between ROS, NF-κB, and the NLRP3 inflammasome. Optineurin can participate in the regulation of the NF-κB signaling pathway. Studies have shown that silencing the optineurin gene in mouse RTEC increased mtROS and led to activation of the NLRP3 inflammasome in a high glucose environment. By enhancing mitophagy, overexpression of the optineurin gene can inhibit the activation of the NLRP3 inflammasome [127]^.^ In addition, a recent study revealed that the suppression of toll-like receptor 9 gene expression in diabetic nephropathy mice can also effectively inhibit the activation of NF-κB and NLRP3 inflammasome pathways, thereby reducing the expression of inflammatory and apoptotic factors [128].

### 5.2. Hypertensive nephropathy

Hypertensive nephropathy is the second leading cause of CKD after DN. Angiotensin II (Ang II), a fundamental element of the renin-angiotensin system, is a crucial regulator of hypertensive nephropathy. By activating the NLRP3 inflammasome, previous studies have suggested that Ang II plays a role in the pathological process of renal fibrosis. A recent study suggested that TGF-β-mediated NLRP3 inflammasome activation may trigger the release of high-mobility group box 1, thereby exacerbating the pathological progression of Ang II-induced renal fibrosis in hypertensive nephropathy. However, further experiments confirmed that TGF-β rather than Ang II-induced NLRP3 protein expression, indicating that there may not be a direct relationship between NLRP3 and Ang II [129]. Another study found that the NLRP3 activity inhibitor MCC950 can lower blood pressure in mice and reduce kidney inflammation, fibrosis, and dysfunction in hypertensive mice [130]. This finding suggests that inhibiting NLRP3 activation may attenuate the progression of hypertensive nephropathy.

Moreover, the intricate interplay between NLRP3 and gut microbiota plays a role in the pathological progression of hypertensive nephropathy. Experimental data indicates that butyric acid can markedly suppress the expression of NLRP3 inflammasome-related proteins triggered by Ang II, thus mitigating the inflammatory response mediated by the NF-κB/IL-1β signaling pathway and decreasing pyroptosis [131]. This ultimately exerts a beneficial effect on hypertensive nephropathy. Looking ahead, interventions aimed at the NLRP3 inflammasome may offer novel therapeutic targets for the treatment of hypertensive nephropathy.

### 5.3. IgA nephropathy

IgA nephropathy(IgAN) is a highly widespread primary glomerular disease worldwide and is regarded as a leading cause of ESRD. The syndrome is characterized by the buildup of immunological complexes mostly consisting of IgA within the glomerular tunica albuginea. The exact cause and pathology of IgAN are still not fully understood, but NLRP3 inflammasomes are seen as promising therapeutic targets [132].

NLRP3 inflammasomes are shown to be significantly linked to the pathological process of IgAN. Renal macrophages and thylakoid cells are implicated in the pathogenesis of IgAN [133]. IgA immune complexes induce mtROS production in macrophages, subsequently activating NLRP3 inflammasomes. IgA immune complexes directly activate NLRP3 inflammasomes in renal parenchymal cells, such as thylakoid cells and renal tubular epithelial cells, leading to a localized renal inflammatory response. Renal tubular injury and interstitial fibrosis are significant factors in the progression of IgAN [22,134]. The expression level of NLRP3 in glomeruli and tubules is significantly elevated in renal biopsy tissues from IgAN patients compared to normal renal tissues [135]. This expression may exhibit an inverse correlation with the severity of renal insufficiency and the quantity of proteinuria. The inhibition of NLRP3 expression has been shown to reverse proteinuria, enhance renal function, and reduce renal lesions in an IgAN model, indicating that the activation of NLRP3 inflammasome is crucial in the pathological progression of this disease [132].

Although previous studies have predominantly indicated that NLRP3 expression in IgAN is concentrated in macrophages, tethered cells and renal tubular epithelial cells, among other cell types, a recent *ex vivo* study unveils a novel role localization of NLRP3 expression in podocytes and suggests that IgA deposition induces NLRP3 expression in podocytes as well as macrophage transdifferentiation [136]. In addition, a study in IgAN patients demonstrated that inhibition of the NLRP3/ASC/caspase-1 signaling pathway by probiotic supplementation significantly ameliorated intestinal dysbiosis and ameliorated IgAN, revealing complex interactions between NLRP3 and intestinal flora in IgA [137]. These changes may influence the immunoregulation and pathological course of IgAN.

### 5.4. Hyperuricemic nephropathy

Hyperuricemic nephropathy is a renal disease directly caused by hyperuricemia. The condition is characterized by decreased renal function, tubulointerstitial fibrosis, and inflammation. Hyperuricemia is now acknowledged as an independent risk factor for an unfavorable prognosis in patients with CKD [138].

The NLRP3 inflammasome is an important player in the development of hyperuricemia nephropathy. Soluble uric acid and its crystals deposited in kidney tissue can act as DAMPs to activate the NLRP3 inflammasome, which in turn triggers the release of inflammatory mediators. Inhibiting NLRP3 activation has been shown to slow the progression of hyperuricemic nephropathy [139]. Hu et al. showed that by disrupting the autophagy-NLRP3 pathway, uric acid-induced pyroptosis could be mitigated effectively, thus preventing the onset of hyperuricemia nephropathy [140]. Wu et al. demonstrated that Shizhifang downregulated the gene and protein expression of the NLRP3 inflammasome by modulating the ROS-TXNIP pathway, thereby reducing renal tubule damage and inflammatory response in hyperuricemia rats [141]. Lin et al. uncovered that Bi Xie Fen Qing Yin decoction could inhibit NLRP3 inflammasome activation by modulating intestinal flora, consequently impeding renal fibrosis in the hyperuricemic nephropathy model [142]. Nonetheless, current research on hyperuricemia nephropathy predominantly relies on animal models and cell studies, underscoring the pressing need for more extensive clinical trials in the future.

### 5.5. Obesity-Associated kidney disease

Obesity-associated kidney disease (OAKD) is a secondary glomerular condition linked to obesity and is a significant risk factor for CKD, with the risk of ESRD increasing with a higher body mass index (BMI) [143].

In a study by Boini et al. in 2014, heightened expression of NLRP3 inflammasome components was observed in damaged podocytes in a mouse model of obesity-induced glomerulopathy from a high-fat diet, indicating activation of the NLRP3 inflammasome [144]. Hou et al. identified that the purinergic 2 × 7 receptor could induce podocyte injury in OAKD by activating the NLRP3 inflammasome, as demonstrated through additional *in vitro* cell studies and *in vivo* mouse models [145]. Furthermore, in obesity, there is an increase in inflammatory mediators such as TNF-α and IL-6 released by adipose tissue, coupled with a decrease in adiponectin levels, resulting in systemic chronic low-grade inflammation. Research has shown that adiponectin can suppress the expression of NLRP3 inflammasome-related factors and alleviate renal podocyte injury induced by free fatty acids *in vitro* [146]. Presently, investigations on NLRP3 in OAKD predominantly concentrate on its downstream components, with additional pathways necessitating further investigation in future studies.

### 5.6. Obstructive nephropathy

The unilateral ureteral obstruction (UUO) model induces increased kidney pressure by obstructing one ureter, resulting in pathological changes resembling those observed in CKD, including inflammation, apoptosis, and fibrosis [147]. This model is widely used in CKD research. Studies have demonstrated a significant upregulation of NLRP3 inflammasome expression in UUO-induced kidney disease, which correlates positively with renal fibrosis severity [148]. Stimulation of renal mitochondria by NLRP3 agonists leads to the generation of ROS, triggering NLRP3 inflammasome assembly and promoting the release of inflammatory mediators [149]. Knocking out the NLRP3 gene in a mouse UUO model resulted in reduced renal injury and fibrosis, underscoring the pivotal role of the NLRP3 inflammasome in UUO-induced kidney fibrosis [149]. However, further confirmation of these findings is necessary through additional clinical trials.

### 5.7. Other chronic kidney diseases

Lupus nephritis (LN) is the most common and severe renal manifestation of systemic lupus erythematosus, classified as an autoimmune glomerulopathy. In systemic lupus erythematosus patients, antinuclear antibodies (such as anti-double-stranded DNA antibodies)bind to glomerular antigens to form immune complexes, which activate the complement system and trigger glomerular inflammation and injury. If left uncontrolled, LN can lead to glomerulosclerosis, tubular atrophy, and renal interstitial fibrosis, ultimately progressing to CKD. Approximately 20%-30% of LN patients will develop ESRD within 10–15 years [120]. The NLRP3 inflammasome is a key contributor to the pathogenesis of LN. Previous research has shown elevated expression of the NLRP3 inflammasome in LN patients, with levels of NLRP3 protein positively correlating with serum creatinine, urine protein, and renal pathology [150]. Animal studies have demonstrated that silencing or inhibiting NLRP3 can significantly reduce renal damage in LN mice. Liu et al. reported that knockout of guanylate-binding protein 5 suppressed NLRP3 inflammasome activation and secretion of IL-1β and IL-18, leading to decreased proteinuria, blood urea nitrogen, and creatinine levels, improved renal pathology, and slowed LN progression [151]. Yang et al. confirmed that magnolol can mitigate LN by enhancing autophagy and suppressing NLRP3 inflammasome activation [152]. Furthermore, early investigations have shown NLRP3 inflammasome activation in uremic patients undergoing dialysis, possibly triggered by mitochondrial dysfunction (Table 1) [153].

**Table 1.** The Role of NLRP3 in CKD.

Disease	Relevant factors	Animal or cell	Role	References
Diabetic Nephropathy	IL-1β↑,IL-18↑,ASC↑, caspase-1↑,NLRP3↑	Diabetic nephropathy mice	Cellular focal death, mitochondrial damage, autophagy, glomerular injury, interstitial fibrosis and proteinuria	[125]
Hypertensive Nephropathy	IL-1β↑,IL-18↑,ASC↑, caspase-1↑,NLRP3↑	Hypertensive nephropathy mice	Kidney damage and fibrosis	[129]
IgA Nephropathy	NLRP3↑,ASC↑,caspase-1↑,IL-1β↑, IL-18↑	NLRP3−/− mice	Proteinuria and improve renal function damage	[132]
Hyperuricemic Nephropathy	NLRP3↑,ASC↑,caspase-1↑, GSDMD↑, ROS↑,TXNIP↑	Hyperuricemic nephropathy rat, RTEC	Cell death, gut flora damage, kidney injury and renal fibrosis	[140–142]
Obesity-Associated Kidney Disease	ASC↑,caspase-1↑,NLRP3↑,Purinergic Receptor P2X 7 R↑	ORG mice	Podocyte injury	[145]
Obstructive Nephropathy	ASC↑,caspase-1↑,IL-1β↑,NLRP3↑	UUO mice,NLRP3−/− mice	Kidney damage and fibrosis	[149]
Lupus Nephritis	NLRP3↑,ASC↑,caspase-1↑,IL-1β↑, IL-18↑	GBP5MRL/*lpr* mice, HK-2 cells	Urine proteinuria, blood urea nitrogen, creatinine levels, renal damage	[151]

**Table 2.** Mechanisms of traditional chinese medicines in the treatment of CKD *via* NLRP3 inflammasome.

Active Ingredients in Chinese Medicines/Complex Chinese Medicines	Source/ composition	Model	Mechanism(s)	References
Astragaloside IV	*Astragalus membranaces*	RTEC	Reduce the generation of ROS to inhibit the activation of the NLRP3 inflammasome.	[158]
PNS	*Panax notoginseng*	CKD Rat、HK-2 cell	Reduce NF-κB p65、NLRP3、IL-1β、IL-18 and inhibit NLRP3 inflammatory vesicle activation and cellular pyroptosis	[160]
Honokiol	*Magnolia*	MRL/*lpr* mouse model	Inhibit of the NLRP3 inflammasome activation and NLRP3/IL-33/ST2 signaling pathway	[164]
Pterostilbene	Plants such as blueberries, grapes and pine trees	Hyperuricemic Nephropathy Rat、NRK-52E cell	Suppress the NLRP3 inflammasome activation and TGF-β-induced epithelial-mesenchymal transition	[122]
Triptolide	*Tripterygium wilfordii*	DN Rat and DN Podocytes	Stimulate Nrf2/HO-1, reduce oxidative stress and ROS generation, and prevent the NLRP3 inflammasome pathway to mitigate cellular apoptosis.	[169]
SMM	*Phellodendrn Bark*、*Rhizoma Atractylodis*、*Achyranthes bidentata* and *Coicis Semen*	Hyperuricemic Nephropathy mouse	Inhibiti the NLRP3, ASC, and caspase-1 expression, along with the release of IL-1β and IL-18, and improve compromised renal function	[171]
BSHX	*Astragalus mongholicus*, *Trigonella foenum-graecum L.*, *Rheum palmatum L.*, *Vaccaria segetalis*, and *Curcuma phaeocaulis Val*	Renal fibrosis cell model and 5/6 nephrectomy rat model	Inhibit inflammation activation mediated by ROS and NLRP3 to prevent renal fibrosis and cellular death.	[13]

## 6. Traditional Chinese medicines

### 6.1. Active ingredients of traditional Chinese medicines

Astragaloside IV is a significant bioactive compound present in the Chinese herbal medicine *Astragalus membranaceus* [154]. It plays a crucial role in retarding the advancement of CKD by mitigating inflammatory responses, reducing apoptosis, and ameliorating oxidative stress [155–157]. Li et al. demonstrated that Astragaloside IV hinders the activation of the NLRP3 inflammasome by diminishing ROS production, consequently suppressing the release of the inflammatory cytokine IL-1β in RTEC. As a result, this action diminishes tubulointerstitial inflammation and decelerates the progression of DN [158]. These findings indicate that astragaloside IV can impede the activation of the NLRP3 inflammasome by improving oxidative stress, as a potential mechanism for managing kidney diseases.

Panax notoginseng saponins (PNS), sourced from *Panax notoginseng*, a traditional Chinese herbal remedy, are known for their significant role in modulating renal fibrosis. Xie et al. demonstrated that PNS effectively reduced blood levels of inflammatory proteins NF-κB p65, NLRP3, IL-1β, and IL-18 in adenine-induced CKD rats while inhibiting the progression of inflammation and fibrosis in renal tissue by modulating gut microbiota and suppressing the activation of renal pro-inflammatory and pro-fibrotic proteins [159]. In a lipopolysaccharide-induced HK-2 cells fibrosis model, PNS attenuated renal fibrosis by suppressing NLRP3 inflammasome activation and cellular pyroptosis. PNS and NLRP3 inflammasome have shown promising results in the treatment of kidney disease through these modes of action [160].

Honokiol is an allyl-substituted biphenyl diphenol molecule that serves as the principal active component of the traditional Chinese medication Magnolia [161]. Recent research indicates that honokiol positively influences the course of CKD through many mechanisms, including anti-inflammatory, antioxidant, and antifibrotic activities [162]. Ma et al. reported in a study utilizing MRL/*lpr* model mice that honokiol inhibited the activation of NLRP3 inflammasomes in the kidneys, thereby mitigating renal damage and pathological alterations in the animals [163]. Moreover, honokiol has demonstrated efficacy in alleviating lupus nephritis by obstructing the NLRP3/IL-33/ST2 signaling pathway, thereby mitigating the abnormal interactions between renal resident macrophages and RTEC [164].

Pterostilbene is a natural dimethoxy analog present in various plant sources like blueberries, grapes, and pine trees [165]. Pterostilbene has been shown to possess renal protective properties by reducing oxidative stress and fibrosis, potentially through modulation of the TGF-β1/Smads signaling pathway [166,167]. Additionally, pterostilbene has emerged as a potent inducer of autophagy in cellular systems. Wang et al. illustrated pterostilbene’s ability to trigger autophagy in a murine model of urate nephropathy, leading to the inhibition of TGF-β-induced NLRP3 inflammasome activation and epithelial-mesenchymal transition, consequently impeding renal fibrosis progression [122]. These findings underscore the therapeutic promise of pterostilbene in managing CKD.

Triptolide is a vital active compound derived from *Tripterygium wilfordii*, a traditional Chinese medicinal plant. Triptolide effectively inhibits the assembly and activation of the NLRP3 inflammasome, leading to a reduction in the expression levels of downstream effector molecules like caspase-1, IL-1β, and IL-18. This inhibition results in decreased renal inflammatory responses [168]. Lv et al. have illustrated that in DN mouse models, activation of the nuclear factor-erythroid 2-related factor 2 (Nrf2)/heme oxygenase-1 pathway can alleviate oxidative stress and reduce ROS production. Furthermore, it can impede the NLRP3 inflammasome pathway, thus alleviating pyroptosis and diabetic nephropathy podocyte injury [169]. Triptolide mitigates oxidative stress and pyroptosis through the Nrf2/ROS/NLRP3 axis, ultimately enhancing renal function and ameliorating histopathological damage in DN mice.

### 6.2. Traditional chinese medicines prescriptions

Few studies have investigated the impact of TCMS prescriptions on regulating the NLRP3 inflammasome in CKD.

Simiao Powder (SMM), a derivative of Ermiao Powder containing *Achyranthes bidentata* and *Coicis Semen*, has shown renal protective properties [170]. A recent study by Shui et al. revealed that SMM may alleviate kidney injury by modulating NLRP3 inflammasome activation. In a mouse model of hyperuricemia induced by potassium oxalate, SMM downregulated the expression of NLRP3, ASC, and caspase-1, as well as the secretion of IL-1β and IL-18. This inhibition of NLRP3 inflammasome activation by SMM ameliorated hyperuricemia, renal dysfunction, and renal histopathological changes induced by PO [171]. These findings highlight the potential of SMM in renal protection.

The Bushen Huoxue (BSHX) prescription is a significant formula utilized in CKD treatment, comprising *Astragalus mongholicus*, *Trigonella foenum-graecum L.*, *Rheum palmatum L.*, *Vaccaria segetalis*, and *Curcuma phaeocaulis Val*. BSHX prescription is commonly used in clinical practice for CKD management, with studies indicating its efficacy in enhancing renal function. Initial animal studies have demonstrated BSHX’s ability to alleviate renal fibrosis in rats post 5/6 nephrectomy [172]. Liao’s latest study showed that BSHX prescription could inhibit ROS/NLRP3 inflammasome activation in 5/6 nephrectomy rats, reduce fibrosis and pyroptosis of HK-2 cells induced by NLRP3 overexpression, and ultimately protect renal function [13]. These findings suggest that BSHX prescription may influence renal oxidative stress and pyroptosis pathways by regulating the NLRP3 inflammasome, thereby offering kidney protection at a molecular level. The potential of BSHX prescription in CKD treatment warrants further investigation (Table 2).

## 7. Synthetic drugs

Since the discovery of the NLRP3 inflammasome, synthetic chemicals targeting this pathway have become a focal point of research. Beyond TCMS extracts and formulations, certain synthetic drugs have demonstrated potential in mitigating CKD by inhibiting NLRP3 inflammasome activation.

Monash Chemical Compound 950 (MCC950), developed by Matt Cooper, is a potent NLRP3 inhibitor that directly interacts with the NLRP3 NACHT domain, blocking ATP hydrolysis and suppressing inflammation assembly and activation. Studies have shown that MCC950 can inhibit NLRP3 activation in *db/db* mice and high-glucose-induced mesangial cells, reducing active caspase-1 and IL-1β levels. Additionally, it lowers serum creatinine, urine albumin-to-creatinine ratio, and neutrophil gelatinase-associated lipocalin, while downregulating TGF-β1, fibronectin, collagen I, and α-SMAD, suggesting a potential therapeutic strategy for diabetic nephropathy. In a cisplatin-induced renal fibrosis model, MCC950 was found to significantly alleviate renal dysfunction, tubular injury, interstitial collagen deposition, and profibrotic factor expression by inhibiting NLRP3 inflammasome activation. This ultimately reduces cisplatin-induced renal fibrosis by mitigating oxidative stress and inflammatory responses [173]. However, some studies have revealed that MCCP50’s inhibition of NLRP3 does not consistently yield renal protective effects in diabetic mice. Instead, it may exacerbate kidney inflammation and damage, including mesangial expansion and glomerulosclerosis [174]. Therefore, the safety of MCC950 in treating kidney diseases requires further investigation.

CY-09, a novel selective NLRP3 inflammasome inhibitor, binds to the ATP-binding motif of the NACHT domain, inhibits NLRP3 ATPase activity, and blocks inflammation activation. It shows promise in preventing CKD progression. *In vitro*, CY-09 dose-dependently inhibits NLRP3 inflammasome activation and reduces caspase-1, IL-18, and IL-1β expression, as well as cell apoptosis. In *db/db* mice, it significantly alleviates DN-induced inflammation, oxidative stress, apoptosis, and fibrosis by inhibiting NLRP3 inflammasome activation [124]. However, research on CY-09 in the field of CKD remains limited.

Phosphoramidon, an endothelin-converting enzyme inhibitor, may indirectly inhibit NLRP3 inflammasome activation to treat CKD. In a rat CKD model, it protected the kidneys by inhibiting ERS in RTEC and inducing autophagy, suppressing NLRP3 inflammasome activation induced by lipopolysaccharide and ATP, lowering serum creatinine, and alleviating pathological damage such as tubular dilation, glomerular atrophy, and interstitial inflammatory cell infiltration [175].

## 8. Conclusions

The NLRP3 inflammasome, a pivotal multi-protein complex in the immune system, has increasingly emerged as a research focus due to its involvement in CKD. Numerous investigations highlight its critical role in initiating and progressing CKD, suggesting its potential as a novel therapeutic target for CKD management. Recent research has explored TCMS for its multi-component, multi-target properties and minimal side effects. This review synthesized evidence from multiple studies, identifying active TCMS compounds—such as astragaloside IV, notoginsenoside, magnolol, pterostilbene, and triptolide—and formulations like Simiao Powder and Bushen Huoxue Prescription—that regulate NLRP3 inflammasome activation to combat CKD. These findings offer valuable insights into the precision clinical application of TCMS in CKD.

Despite the promising research potential of traditional Chinese medicines’ active ingredients, several challenges persist. Current NLRP3-related studies predominantly focus on animal models and *in vitro* experiments, with a paucity of high-quality clinical trials on TCMS components and formulas. Noteworthy, compounds such as astrgaloside IV and notoginsenoside have exhibited remarkable preclinical efficacy; however, interindividual variability in metabolism and response to TCMS components can lead to inconsistent regulation of NLRP3 inflammasome in certain patients. The absence of compound-specific pharmacokinetic data (e.g., bioavailability, tissue distribution) hinders dose optimization and safety assessment. Additionally, many herbal components suffer from unstable concentrations, low purity, non-standardized extraction processes, poor bioavailability, and unclear mechanisms/safety profiles, limiting clinical translation.The complexity of TCMS composition further poses challenges. TCMS from different regions/batches may vary in constituents, complicating standardization and quality control. In the absence of uniform quality metrics, the efficacy of TCMS remains inconsistent, affecting clinical application. Moreover, the NLRP3 inflammasome activation mechanism is incompletely understood, involving complex crosstalk of multiple signaling pathways that hinder precise regulation. Most studies focus on single pathways, failing to fully reveal traditional Chinese medicine’s multi-target/multi-pathway regulatory effects. Additionally, a lack of validation tools (e.g., gene knockout models, specific antagonists) undermines evidence for TCMS-target specificity.Importantly, given the complex pathophysiology and heterogeneous subtypes of CKD, findings from studies targeting specific kidney diseases cannot extrapolate to all CKD forms. Large-sample, multicenter clinical and experimental studies are urgently needed to validate the efficacy and safety of TCMS components/formulas across CKD subtypes - a critical research priority.

Future research should prioritize multicenter, prospective, stratified randomized controlled trials (stratified by CKD etiology, pathology, and NLRP3 genotype) under TCMS theoretical guidance, exploring the efficacy and safety of TCMS in CKD patients and its mechanisms in regulating NLRP3 inflammasome activation. Technologies like CRISPR-Cas9 should be used to identify TCMS components directly targeting NLRP3, while UPLC-Q-TOF-MS can establish fingerprint maps of NLRP3-regulating TCMS components. *In vivo* imaging (e.g., two-photon microscopy) may visualize TCMS component distribution in kidneys and colocalization with NLRP3. Constructing a ‘formula-components-targets-pathways’ research framework will analyze synergistic/antagonistic effects of TCMS multi-components on NLRP3 inflammasome. Enhancing TCMS quality control standards and developing targeted delivery systems may increase drug concentration/duration in target organs, improving efficacy. Single-cell sequencing and spatial transcriptomics can reveal key TCMS regulatory targets and inflammatory dynamics across cell types and time points, while reverse validation (e.g., blockers, gene knockout) will clarify mechanisms of TCMS formulas/components in targeting NLRP3 inflammasome. These efforts are essential for advancing CKD-targeted drug development.

## References

[1] Kalantar-Zadeh K, Jafar TH, Nitsch D, et al. Chronic kidney disease. Lancet. 2021;398(10302):786–802. doi:10.1016/S0140-6736(21)00519-5. 34175022

[2] Francis A, Harhay MN, Ong ACM, et al. Chronic kidney disease and the global public health agenda: an international consensus. Nat Rev Nephrol. 2024;20(7):473–485. doi:10.1038/s41581-024-00820-6. 38570631

[3] Kalyesubula R, Luyckx VA. Managing risk factors and early intervention for chronic kidney disease. Nat Rev Nephrol. 2025;21(3):149–150. doi:10.1038/s41581-025-00930-9. 39814983

[4] Wang L, Xu X, Zhang M, et al. Prevalence of chronic kidney disease in china: results from the sixth china chronic disease and risk factor surveillance. JAMA Intern Med. 2023;183(4):298–310. doi:10.1001/jamainternmed.2022.6817. 36804760 PMC9941971

[5] Lamkanfi M, Dixit VM. The inflammasome turns 15. Nature. 2017;548(7669):534–535. doi:10.1038/548534a. 28858314

[6] Sharma BR, Kanneganti T-D. NLRP3 inflammasome in cancer and metabolic diseases. Nat Immunol. 2021;22(5):550–559. doi:10.1038/s41590-021-00886-5. 33707781 PMC8132572

[7] Toldo S, Abbate A. The role of the NLRP3 inflammasome and pyroptosis in cardiovascular diseases. Nat Rev Cardiol. 2024;21(4):219–237. doi:10.1038/s41569-023-00946-3. 37923829 PMC11550901

[8] Xu W, Huang Y, Zhou R. NLRP3 inflammasome in neuroinflammation and central nervous system diseases. Cell Mol Immunol. 2025;22(4):341–355. doi:10.1038/s41423-025-01275-w. 40075143 PMC11955557

[9] Henedak NT, El-Abhar HS, Soubh AA, et al. NLRP3 Inflammasome: a central player in renal pathologies and nephropathy. Life Sci. 2024;351:122813. doi:10.1016/j.lfs.2024.122813. 38857655

[10] Giuliani KTK, Grivei A, Nag P, et al. Hypoxic human proximal tubular epithelial cells undergo ferroptosis and elicit an NLRP3 inflammasome response in CD1c(+) dendritic cells. Cell Death Dis. 2022;13(8):739. doi:10.1038/s41419-022-05191-z. 36030251 PMC9420140

[11] Yong J, Hu H, Zhanjun J, et al. Therapy Targeted to the NLRP3 Inflammasome in Chronic Kidney Disease. Kidney Dis (Basel). 2024;10(5):369–383. doi:10.1159/000539496. PMC11488838 39430292

[12] Jin J, Zhang M. Exploring the role of NLRP3 inflammasome in diabetic nephropathy and the advancements in herbal therapeutics. *Front Endocrinol (Lausanne)*. 2024;15:1397301. doi:10.3389/fendo.2024.1397301. PMC11299242 39104818

[13] Lin L, Pengyu T, Qiming X, et al. Bushen Huoxue formula protects against renal fibrosis and pyroptosis in chronic kidney disease by inhibiting ROS/NLRP3-mediated inflammasome activation. Renal Failure. 2024;46(1):2354444. doi:10.1080/0886022X.2024.2354444. PMC11132749 38785272

[14] Hu L, Li L, Che H, et al. Huanglian Decoction Treats HenochSchonlein Purpura Nephritis by Inhibiting NF-κB/NLRP3 Signaling Pathway and Reducing Renal IgA Deposition. An Acad Bras Cienc. 2024;96(1):e20220970. doi:10.1590/0001-3765202420220970. 38597498

[15] Wen H, Ting JP, O’Neill LA. A role for the NLRP3 inflammasome in metabolic diseases–did Warburg miss inflammation? Nat Immunol. 2012;13(4):352–357. doi:10.1038/ni.2228. 22430788 PMC4090390

[16] Fu J, Wu H. Structural mechanisms of NLRP3 inflammasome assembly and activation. Annu Rev Immunol. 2023;41(1):301–316. doi:10.1146/annurev-immunol-081022-021207. 36750315 PMC10159982

[17] Lu A, Magupalli VG, Ruan J, et al. Unified polymerization mechanism for the assembly of ASC-dependent inflammasomes. Cell. 2014;156(6):1193–1206. doi:10.1016/j.cell.2014.02.008. 24630722 PMC4000066

[18] Martinon F, Burns K, Tschopp J. The inflammasome: a molecular platform triggering activation of inflammatory caspases and processing of proIL-beta. Mol Cell. 2002;10(2):417–426. doi:10.1016/s1097-2765(02)00599-3. 12191486

[19] Wang K, Sun Q, Zhong X, et al. Structural mechanism for GSDMD targeting by autoprocessed caspases in pyroptosis. Cell. 2020;180(5):941–955.e920. doi:10.1016/j.cell.2020.02.002. 32109412

[20] Guo H, Callaway JB, Ting JPY. Inflammasomes: mechanism of action, role in disease, and therapeutics. Nat Med. 2015;21(7):677–687. doi:10.1038/nm.3893. 26121197 PMC4519035

[21] Malik A, Kanneganti TD. Inflammasome activation and assembly at a glance. J Cell Sci. 2017;130(23):3955–3963. doi:10.1242/jcs.207365. 29196474 PMC5769591

[22] Swanson KV, Deng M, Ting JP. The NLRP3 inflammasome: molecular activation and regulation to therapeutics. Nat Rev Immunol. 2019;19(8):477–489. doi:10.1038/s41577-019-0165-0. 31036962 PMC7807242

[23] Kodi T, Sankhe R, Gopinathan A, et al. New insights on NLRP3 inflammasome: mechanisms of activation, inhibition, and epigenetic regulation. J Neuroimmune Pharmacol. 2024;19(1):7. doi:10.1007/s11481-024-10101-5. 38421496 PMC10904444

[24] Xu J, Núñez G. The NLRP3 inflammasome: activation and regulation. Trends Biochem Sci. 2023;48(4):331–344. doi:10.1016/j.tibs.2022.10.002. 36336552 PMC10023278

[25] Gong T, Liu L, Jiang W, et al. DAMP-sensing receptors in sterile inflammation and inflammatory diseases. Nat Rev Immunol. 2020;20(2):95–112. doi:10.1038/s41577-019-0215-7. 31558839

[26] Nagar A, Bharadwaj R, Shaikh MOF, et al. What are NLRP3-ASC specks? an experimental progress of 22 years of inflammasome research. Front Immunol. 2023;14:1188864. volume2023. doi:10.3389/fimmu.2023.1188864. 37564644 PMC10411722

[27] Zheng R, Yan Y, Dai S, et al. ASC specks exacerbate α‑synuclein pathology via amplifying NLRP3 inflammasome activities. J Neuroinflammation. 2023;20(1):26. doi:10.1186/s12974-023-02709-w. 36740674 PMC9899382

[28] Dinarello CA. The C3a receptor, caspase-1, and release of IL-1β. Blood. 2013;122(20):3394–3395. doi:10.1182/blood-2013-08-518282. 24235127 PMC3829112

[29] Huang Y, Xu W, Zhou R. NLRP3 inflammasome activation and cell death. Cell Mol Immunol. 2021;18(9):2114–2127. doi:10.1038/s41423-021-00740-6. 34321623 PMC8429580

[30] Liu Z, Wang C, Yang J, et al. Caspase-1 engages full-length gasdermin D through two distinct interfaces that mediate caspase recruitment and substrate cleavage. Immunity. 2020;53(1):106–114.e5. e105. doi:10.1016/j.immuni.2020.06.007. 32553275 PMC7382298

[31] ChangYan L, Chen G, JiangMing W, et al. Investigating the inflammatory mechanism of notoginsenoside R1 in Diabetic nephropathy via ITGB8 based on network pharmacology and experimental validation. Mol Med. 2024;30(1):277. doi:10.1186/s10020-024-01055-8. PMC11674371 39725889

[32] Shahzad K, Fatima S, Khawaja H, et al. Podocyte-specific NLRP3 inflammasome activation promotes diabetic kidney disease. Kidney Int. 2022;102(4):766–779. doi:10.1016/j.kint.2022.06.010. 35779608

[33] Zamyatina A, Heine H. Corrigendum: lipopolysaccharide Recognition in the crossroads of TLR4 and Caspase-4/11 mediated inflammatory pathways. Front Immunol. 2021;12:649442. doi:10.3389/fimmu.2021.649442. 33584736 PMC7876549

[34] Zhu F, Ma J, Li W, et al. The orphan receptor Nur77 binds cytoplasmic LPS to activate the non-canonical NLRP3 inflammasome. Immunity. 2023;56(4):753–767.e758. doi:10.1016/j.immuni.2023.03.003. 37001519

[35] Orzalli MH. An orphan no more: nur77 senses cytosolic LPS. Immunity. 2023;56(4):742–744. doi:10.1016/j.immuni.2023.03.012. 37044063

[36] Kang S, Fernandes-Alnemri T, Rogers C, et al. Caspase-8 scaffolding function and MLKL regulate NLRP3 inflammasome activation downstream of TLR3. Nat Commun. 2015;6(1):7515. doi:10.1038/ncomms8515. 26104484 PMC4480782

[37] Chung H, Vilaysane A, Lau A, et al. NLRP3 regulates a non-canonical platform for caspase-8 activation during epithelial cell apoptosis. Cell Death Differ. 2016;23(8):1331–1346. doi:10.1038/cdd.2016.14. 26891693 PMC4947664

[38] Zheng M, Kanneganti TD. The regulation of the ZBP1-NLRP3 inflammasome and its implications in pyroptosis, apoptosis, and necroptosis (PANoptosis). Immunol Rev. 2020;297(1):26–38. doi:10.1111/imr.12909. 32729116 PMC7811275

[39] Gong T, Yang Y, Jin T, et al. Orchestration of NLRP3 inflammasome activation by ion fluxes. Trends Immunol. 2018;39(5):393–406. doi:10.1016/j.it.2018.01.009. 29452983

[40] Han Y, Xu X, Tang C, et al. ROS promote tubular injury in diabetic nephropathy: the role of the mitochondrial ros-*TXNIP*-NLRP3 biological axis. Redox Biol. 2018;16:32–46. doi:10.1016/j.redox.2018.02.013. 29475133 PMC5842313

[41] Zhou Y, Tong Z, Jiang S, et al. The roles of endoplasmic reticulum in NLRP3 inflammasome Activation. Cells. 2020;9(5):1219. doi:10.3390/cells9051219. 32423023 PMC7291288

[42] Li Z, Yao Y-X, Lu X, et al. Short-term respiratory cadmium exposure partially activates pulmonary NLRP3 inflammasome by inducing ferroptosis in mice. Ecotoxicol Environ Saf. 2024;285:117106. doi:10.1016/j.ecoenv.2024.117106. 39326353

[43] Malireddi RKS, Bynigeri RR, Mall R, et al. Whole-genome CRISPR screen identifies RAVER1 as a key regulator of RIPK1-mediated inflammatory cell death, PANoptosis. iScience. 2023;26(6):106938. doi:10.1016/j.isci.2023.106938. 37324531 PMC10265528

[44] Zhou X, Ji S, Chen L, et al. Gut microbiota dysbiosis in hyperuricaemia promotes renal injury through the activation of NLRP3 inflammasome. Microbiome. 2024;12(1):109. doi:10.1186/s40168-024-01826-9. 38907332 PMC11191305

[45] Muñoz-Planillo R, Kuffa P, Martínez-Colón G, et al. Is the common trigger of NLRP3 inflammasome activation by bacterial toxins and particulate matter. Immunity. 2013;38(6):1142–1153. ^+^ doi:10.1016/j.immuni.2013.05.016. 23809161 PMC3730833

[46] Seoane PI, Beswick JA, Leach AG, et al. Squaramides enhance NLRP3 inflammasome activation by lowering intracellular potassium. Cell Death Discov. 2023;9(1):469. doi:10.1038/s41420-023-01756-9. 38129373 PMC10739973

[47] He Y, Zeng MY, Yang D, et al. *NEK7* is an essential mediator of NLRP3 activation downstream of potassium efflux. Nature. 2016;530(7590):354–357. doi:10.1038/nature16959. 26814970 PMC4810788

[48] Sharif H, Wang L, Wang WL, et al. Structural mechanism for *NEK7*-licensed activation of NLRP3 inflammasome. Nature. 2019;570(7761):338–343. doi:10.1038/s41586-019-1295-z. 31189953 PMC6774351

[49] Xu J, Zhang L, Duan Y, et al. *NEK7* phosphorylation amplifies NLRP3 inflammasome activation downstream of potassium efflux and gasdermin D. Sci Immunol. 2025;10(103):eadl2993. doi:10.1126/sciimmunol.adl2993. 39752537 PMC12020992

[50] Martín-Sánchez F, Compan V, Peñín-Franch A, et al. ASC oligomer favors caspase-1CARD domain recruitment after intracellular potassium efflux. Journal of Cell Biology. 2023;222(8):e202003053. doi:10.1083/jcb.202003053. 37402211 PMC10318405

[51] Groß CJ, Mishra R, Schneider KS, et al. K(+) efflux-independent NLRP3 inflammasome activation by small molecules targeting mitochondria. Immunity. 2016;45(4):761–773. doi:10.1016/j.immuni.2016.08.010. 27692612

[52] Holley CL, Emming S, Monteleone MM, et al. The septin modifier, forchlorfenuron, activates NLRP3 via a potassium-independent mitochondrial axis. Cell Chem Biol. 2024;31(5):962–972.e4. e964. doi:10.1016/j.chembiol.2024.04.012. 38759620

[53] He X, Peng Y, Huang S, et al. Blood brain barrier-crossing delivery of felodipine nanodrug ameliorates anxiety-like behavior and cognitive impairment in Alzheimer’s disease. Adv Sci. 2024;11(34):2401731. doi:10.1002/advs.202401731. PMC11425895 38981028

[54] Jäger E, Murthy S, Schmidt C, et al. Calcium-sensing receptor-mediated NLRP3 inflammasome response to calciprotein particles drives inflammation in rheumatoid arthritis. Nat Commun. 2020;11(1):4243. doi:10.1038/s41467-020-17749-6. 32843625 PMC7447633

[55] Murakami T, Ockinger J, Yu J, et al. Critical role for calcium mobilization in activation of the NLRP3 inflammasome. Proc Natl Acad Sci U S A. 2012;109(28):11282–11287. doi:10.1073/pnas.1117765109. 22733741 PMC3396518

[56] Kang H, Choi SW, Kim JY, et al. ER-to-lysosome Ca(2+) refilling followed by K(+) efflux-coupled store-operated Ca(2+) entry in inflammasome activation and metabolic inflammation. Elife. 2024;12:RP87561. doi:10.7554/eLife.87561. PMC11219040 38953285

[57] Kendall RL, Holian A. Lysosomal BK channels facilitate silica-induced inflammation in macrophages. Inhal Toxicol. 2024;36(1):31–43. doi:10.1080/08958378.2024.2305112. 38261520 PMC11080613

[58] Ran L, Ye T, Erbs E, et al. KCNN4 links PIEZO-dependent mechanotransduction to NLRP3 inflammasome activation. Sci Immunol. 2023;8(90):eadf4699. doi:10.1126/sciimmunol.adf4699. 38134241

[59] Katsnelson MA, Rucker LG, Russo HM, et al. K^+^ efflux agonists induce NLRP3 inflammasome activation independently of Ca^2+^ signaling. J Immunol. 2015;194(8):3937–3952. doi:10.4049/jimmunol.1402658. 25762778 PMC4390495

[60] Hsueh-Hsiao W, Hsin-Chung C-RH, Hsin-An L, et al. Magnesium-enriched deep-sea water inhibits NLRP3 inflammasome activation and dampens inflammation. Heliyon. 2024;10(15):e35136. doi:10.1016/j.heliyon.2024.e35136. PMC11327587 39157306

[61] Li C, Chen M, He X, et al. A mini-review on ion fluxes that regulate NLRP3 inflammasome activation. Acta Biochim Biophys Sin (Shanghai). 2021;53(2):131–139. doi:10.1093/abbs/gmaa155. 33355638

[62] Pitzer A, Elijovich F, Laffer CL, et al. DC ENaC-dependent inflammasome activation contributes to salt-sensitive hypertension. Circ Res. 2022;131(4):328–344. doi:10.1161/CIRCRESAHA.122.320818. 35862128 PMC9357159

[63] Tang T, Lang X, Xu C, et al. CLICs-dependent chloride efflux is an essential and proximal upstream event for NLRP3 inflammasome activation. Nat Commun. 2017;8(1):202. doi:10.1038/s41467-017-00227-x. 28779175 PMC5544706

[64] Galvan DL, Green NH, Danesh FR. The hallmarks of mitochondrial dysfunction in chronic kidney disease. Kidney Int. 2017;92(5):1051–1057. doi:10.1016/j.kint.2017.05.034. 28893420 PMC5667560

[65] Tang C, Cai J, Yin XM, et al. Mitochondrial quality control in kidney injury and repair. Nat Rev Nephrol. 2021;17(5):299–318. doi:10.1038/s41581-020-00369-0. 33235391 PMC8958893

[66] Hsu W-H, Hua K-F, Tuan L-H, et al. Compound K inhibits priming and mitochondria-associated activating signals of NLRP3 inflammasome in renal tubulointerstitial lesions. Nephrology Dialysis Transplantation. 2020;35(1):74–85. 10.1093/ndt/gfz073 31065699

[67] Zhou R, Yazdi AS, Menu P, et al. A role for mitochondria in NLRP3 inflammasome activation. Nature. 2011;469(7329):221–225. doi:10.1038/nature09663. 21124315

[68] Nam BY, Jhee JH, Park J, et al. PGC-1α inhibits the NLRP3 inflammasome via preserving mitochondrial viability to protect kidney fibrosis. Cell Death Dis. 2022;13(1):31. doi:10.1038/s41419-021-04480-3. 35013155 PMC8748677

[69] Billingham LK, Stoolman JS, Vasan K, et al. Mitochondrial electron transport chain is necessary for NLRP3 inflammasome activation. Nat Immunol. 2022;23(5):692–704. doi:10.1038/s41590-022-01185-3. 35484407 PMC9098388

[70] Choi E-H, Park S-J. *TXNIP*: a key protein in the cellular stress response pathway and a potential therapeutic target. Exp Mol Med. 2023;55(7):1348–1356. doi:10.1038/s12276-023-01019-8. 37394581 PMC10393958

[71] Fatma E, George SGS, Eman M-K, et al. Diltiazem mitigates acute liver injury by targeting NFκB-*TXNIP*/NLRP3 axis in Rats: new insights beyond calcium channel blockade. Int Immunopharmacol. 2024;143(Pt 2):113460. doi:10.1016/j.intimp.2024.113460. 39514911

[72] Pan M, Zhang F, Qu K, et al. *TXNIP*: a double-edged sword in disease and therapeutic outlook. Oxid Med Cell Longev. 2022;2022(1):7805115. doi:10.1155/2022/7805115. 35450411 PMC9017576

[73] Zhou R, Tardivel A, Thorens B, et al. Thioredoxin-interacting protein links oxidative stress to inflammasome activation. Nat Immunol. 2010;11(2):136–140. doi:10.1038/ni.1831. 20023662

[74] Dai X, Liao R, Liu C, et al. Epigenetic regulation of *TXNIP*-mediated oxidative stress and NLRP3 inflammasome activation contributes to SAHH inhibition-aggravated diabetic nephropathy. Redox Biol. 2021;45:102033. doi:10.1016/j.redox.2021.102033. 34119876 PMC8209273

[75] Wen Y, Liu Y-R, Tang T-T, et al. mROS-*TXNIP* axis activates NLRP3 inflammasome to mediate renal injury during ischemic AKI. Int J Biochem Cell Biol. 2018;98:43–53. doi:10.1016/j.biocel.2018.02.015. 29477360

[76] Abais JM, Xia M, Li G, et al. Nod-like receptor protein 3 (NLRP3) inflammasome activation and podocyte injury via Thioredoxin-Interacting Protein (*TXNIP*) during hyperhomocysteinemia. J Biol Chem. 2014;289(39):27159–27168. * doi:10.1074/jbc.M114.567537. 25138219 PMC4175351

[77] Moon J-S, Nakahira K, Chung K-P, et al. RETRACTED ARTICLE: NOX4-dependent fatty acid oxidation promotes NLRP3 inflammasome activation in macrophages. Nat Med. 2016;22(9):1002–1012. doi:10.1038/nm.4153. 27455510 PMC5204248

[78] Sokolovska A, Becker CE, Ip WKE, et al. Activation of caspase-1 by the NLRP3 inflammasome regulates the NADPH oxidase NOX2 to control phagosome function. Nat Immunol. 2013;14(6):543–553. doi:10.1038/ni.2595. 23644505 PMC3708594

[79] Vermot A, Petit-Härtlein I, Smith SME, et al. NADPH oxidases (NOX): an overview from discovery, molecular mechanisms to physiology and pathology. Antioxidants (Basel). 2021;10(6):890. doi:10.3390/antiox10060890. PMC8228183 34205998

[80] Wu M, Han W, Song S, et al. NLRP3 deficiency ameliorates renal inflammation and fibrosis in diabetic mice. Mol Cell Endocrinol. 2018;478:115–125. doi:10.1016/j.mce.2018.08.002. 30098377

[81] Duoduo Z, Pengmin J, Ran S, et al. Ginsenoside Rg1 attenuates LPS-induced chronic renal injury by inhibiting NOX4-NLRP3 signaling in mice. Biomed Pharmacother. 2022;150:112936. doi:10.1016/j.biopha.2022.112936. 35421784

[82] Riley JS, Tait SW. Mitochondrial DNA in inflammation and immunity. EMBO Rep. 2020;21(4):e49799. doi:10.15252/embr.201949799. 32202065 PMC7132203

[83] Zhong Z, Liang S, Sanchez-Lopez E, et al. New mitochondrial DNA synthesis enables NLRP3 inflammasome activation. Nature. 2018;560(7717):198–203. doi:10.1038/s41586-018-0372-z. 30046112 PMC6329306

[84] Meyer-Schwesinger C. Lysosome function in glomerular health and disease. Cell Tissue Res. 2021;385(2):371–392. doi:10.1007/s00441-020-03375-7. 33433692 PMC8523507

[85] Xu H, Ren D. Lysosomal Physiology. Annu Rev Physiol. 2015;77(1):57–80. doi:10.1146/annurev-physiol-021014-071649. 25668017 PMC4524569

[86] Chevriaux A, Pilot T, Derangère V, et al. Cathepsin B is required for NLRP3 inflammasome activation in macrophages, through NLRP3 interaction. Front Cell Dev Biol. 2020;8:167. doi:10.3389/fcell.2020.00167. 32328491 PMC7162607

[87] Cocchiaro P, De Pasquale V, Della Morte R, et al. The Multifaceted role of the lysosomal protease cathepsins in kidney disease. Front Cell Dev Biol. 2017;5:114. doi:10.3389/fcell.2017.00114. 29312937 PMC5742100

[88] Man SM, Kanneganti TD. Regulation of lysosomal dynamics and autophagy by CTSB/cathepsin B. Autophagy. 2016;12(12):2504–2505. doi:10.1080/15548627.2016.1239679. 27786577 PMC5173259

[89] Orlowski GM, Colbert JD, Sharma S, et al. Multiple cathepsins promote pro–IL-1β synthesis and NLRP3-Mediated IL-1β activation. J Immunol. 2015;195(4):1685–1697. doi:10.4049/jimmunol.1500509. 26195813 PMC4530060

[90] Zheng L, Mei W, Zhou J, et al. Fluorofenidone attenuates renal fibrosis by inhibiting lysosomal cathepsin‑mediated NLRP3 inflammasome activation. Exp Ther Med. 2024;27(4):142. doi:10.3892/etm.2024.12430. 38476910 PMC10928820

[91] Bussi C, Heunis T, Pellegrino E, et al. Lysosomal damage drives mitochondrial proteome remodelling and reprograms macrophage immunometabolism. Nat Commun. 2022;13(1):7338. doi:10.1038/s41467-022-34632-8. 36443305 PMC9705561

[92] Hou Y, He H, Ma M, et al. Apilimod activates the NLRP3 inflammasome through lysosome-mediated mitochondrial damage. Front Immunol. 2023;14:1128700. doi:10.3389/fimmu.2023.1128700. 37359517 PMC10285205

[93] Wu D, Huang L-F, Chen X-C, et al. Research progress on endoplasmic reticulum homeostasis in kidney diseases. Cell Death Dis. 2023;14(7):473. doi:10.1038/s41419-023-05905-x. 37500613 PMC10374544

[94] Li W, Cao T, Luo C, et al. Crosstalk between ER stress, NLRP3 inflammasome, and inflammation. Appl Microbiol Biotechnol. 2020;104(14):6129–6140. doi:10.1007/s00253-020-10614-y. 32447438

[95] Elwakiel A, Mathew A, Isermann B. The role of endoplasmic reticulum-mitochondria-associated membranes in diabetic kidney disease. Cardiovasc Res. 2024;119(18):2875–2883. doi:10.1093/cvr/cvad190. 38367274

[96] Hu Z-C, Wang B, Zhou X-G, et al. Golgi apparatus-targeted photodynamic therapy for enhancing tumor immunogenicity by eliciting NLRP3 protein-dependent pyroptosis. ACS Nano. 2023;17(21):21153–21169. doi:10.1021/acsnano.3c05005. 37921421

[97] Zhang Z, Meszaros G, He W-T, et al. Protein kinase D at the Golgi controls NLRP3 inflammasome activation. J Exp Med. 2017;214(9):2671–2693. doi:10.1084/jem.20162040. 28716882 PMC5584123

[98] Cuevas S, Pelegrín P. Pyroptosis and redox balance in kidney diseases. Antioxid Redox Signal. 2021;35(1):40–60. doi:10.1089/ars.2020.8243. 33559516

[99] Jinwen L, Ao C, Kai C, et al. New Insights into the mechanisms of pyroptosis and implications for diabetic kidney disease. Int J Mol Sci. 2020;21(19):7057. 32992874 10.3390/ijms21197057 PMC7583981

[100] Kim YG, Kim S-M, Kim K-P, et al. The role of inflammasome-dependent and inflammasome-independent NLRP3 in the kidney. Cells. 2019;8(11):1389. doi:10.3390/cells8111389. 31694192 PMC6912448

[101] Liao L, Tao P, Xu Q, et al. Bushen Huoxue formula protects against renal fibrosis and pyroptosis in chronic kidney disease by inhibiting ROS/NLRP3-mediated inflammasome activation. Ren Fail. 2024;46(1):2354444. doi:10.1080/0886022X.2024.2354444. 38785272 PMC11132749

[102] Yaqiu W, Thirumala-Devi K. From pyroptosis, apoptosis and necroptosis to PANoptosis: a mechanistic compendium of programmed cell death pathways. Comput Struct Biotechnol J. 2021;19:4641–4657. 34504660 10.1016/j.csbj.2021.07.038 PMC8405902

[103] Kaczmarek A, Vandenabeele P, Krysko DV. Necroptosis: the release of damage-associated molecular patterns and its physiological relevance. Immunity. 2013;38(2):209–223. doi:10.1016/j.immuni.2013.02.003. 23438821

[104] Dixon SJ, Lemberg KM, Lamprecht MR, et al. Ferroptosis: an iron-dependent form of nonapoptotic cell death. Cell. 2012;149(5):1060–1072. doi:10.1016/j.cell.2012.03.042. 22632970 PMC3367386

[105] Zhou L, Xue X, Hou Q, et al. Targeting ferroptosis attenuates interstitial inflammation and kidney fibrosis. Kidney Dis (Basel). 2022;8(1):57–71. doi:10.1159/000517723. 35224007 PMC8820137

[106] Zhang B, Chen X, Ru F, et al. Liproxstatin-1 attenuates unilateral ureteral obstruction-induced renal fibrosis by inhibiting renal tubular epithelial cells ferroptosis. Cell Death Dis. 2021;12(9):843. doi:10.1038/s41419-021-04137-1. 34511597 PMC8435531

[107] Zhao Z, Wu J, Xu H, et al. XJB-5-131 inhibited ferroptosis in tubular epithelial cells after ischemia-reperfusion injury. Cell Death Dis. 2020;11(8):629. doi:10.1038/s41419-020-02871-6. 32796819 PMC7429848

[108] Chen X, Kang R, Kroemer G, et al. Ferroptosis in infection, inflammation, and immunity. J Exp Med. 2021;218(6):e20210518. doi:10.1084/jem.20210518. PMC8126980 33978684

[109] Wen Y, Zhao C, Chen J, et al. Gandouling regulates ferroptosis and improves neuroinflammation in Wilson’s disease through the LCN2/NLRP3 signaling pathway. J Inflamm Res. 2024;17:5599–5618. doi:10.2147/JIR.S465341. 39193124 PMC11348929

[110] Kim JW, Nam SA, Koh E-S, et al. The Impairment of Endothelial Autophagy Accelerates Renal Senescence by Ferroptosis and NLRP3 Inflammasome Signaling Pathways with the Disruption of Endothelial Barrier. *Antioxidants (Basel)*. 2024;13(8):886. doi:10.3390/antiox13080886. PMC11351978 39199133

[111] Forcina GC, Dixon SJ. GPX4 at the crossroads of lipid homeostasis and ferroptosis. Proteomics. 2019;19(18):e1800311. doi:10.1002/pmic.201800311. 30888116

[112] Zhou Y, Zhou H, Hua L, et al. Verification of ferroptosis and pyroptosis and identification of PTGS2 as the hub gene in human coronary artery atherosclerosis. Free Radic Biol Med. 2021;171:55–68. doi:10.1016/j.freeradbiomed.2021.05.009. 33974977

[113] Meijers BK, Evenepoel P. The gut-kidney axis: indoxyl sulfate, p-cresyl sulfate and CKD progression. Nephrol Dial Transplant. 2011;26(3):759–761. doi:10.1093/ndt/gfq818. 21343587

[114] Pan H, Jian Y, Wang F, et al. NLRP3 and gut microbiota homeostasis: progress in research. Cells. 2022;11(23):3758. doi:10.3390/cells11233758. 36497018 PMC9739202

[115] Long C, Zhang C, Xie Y. Study on the mechanism of hirudin multi target delaying renal function decline in chronic kidney disease based on the "gut-kidney axis" theory. Naunyn Schmiedebergs Arch Pharmacol. 2024;397(10):7951–7962. doi:10.1007/s00210-023-02888-6. 38758227 PMC11450085

[116] Pan S, Jiang S-S, Li R, et al. Hong Guo Ginseng Guo (HGGG) protects against kidney injury in diabetic nephropathy by inhibiting NLRP3 inflammasome and regulating intestinal flora. Phytomedicine. 2024;132:155861. doi:10.1016/j.phymed.2024.155861. 39024672

[117] Tian X, Zeng Y, Tu Q, et al. Butyrate alleviates renal fibrosis in CKD by regulating NLRP3-mediated pyroptosis via the STING/NF-κB/p65 pathway. Int Immunopharmacol. 2023;124(Pt B):111010. doi:10.1016/j.intimp.2023.111010. 37852118

[118] Ramos CI, Armani RG, Canziani MEF, et al. Effect of prebiotic (fructooligosaccharide) on uremic toxins of chronic kidney disease patients: a randomized controlled trial. Nephrol Dial Transplant. 2019;34(11):1876–1884. doi:10.1093/ndt/gfy171. 29939302

[119] Wang J, Jiang M, Li X, et al. Inulin supplementation alleviates ochratoxin a-induced kidney injury through modulating intestinal microbiota. J Agric Food Chem. 2024;72(33):18682–18696. doi:10.1021/acs.jafc.4c04382. 39135376

[120] Lichtnekert J, Anders H-J. Lupus nephritis-related chronic kidney disease. Nat Rev Rheumatol. 2024;20(11):699–711. doi:10.1038/s41584-024-01158-w. 39317803

[121] Lin T-A, Wu VC-C, Wang C-Y. Autophagy in chronic kidney diseases. Cells. 2019;8(1):61. doi:10.3390/cells8010061. 30654583 PMC6357204

[122] Wang Y-J, Chen Y-Y, Hsiao C-M, et al. Induction of autophagy by pterostilbene contributes to the prevention of renal fibrosis via attenuating NLRP3 inflammasome activation and epithelial-mesenchymal transition. Front Cell Dev Biol. 2020;8:436. volume2020. doi:10.3389/fcell.2020.00436. 32582712 PMC7283393

[123] Ding H, Li J, Li Y, et al. MicroRNA-10 negatively regulates inflammation in diabetic kidney via targeting activation of the NLRP3 inflammasome. Mol Ther. 2021;29(7):2308–2320. doi:10.1016/j.ymthe.2021.03.012. 33744467 PMC8261077

[124] Ming Y, Li Z. The selective NLRP3-inflammasome inhibitor CY-09 ameliorates kidney injury in diabetic nephropathy by inhibiting NLRP3- inflammasome activation. Curr Med Chem. 2023;30(28):3261–3270. 36154582 10.2174/0929867329666220922104654

[125] Gu C, Liu S, Wang H, et al. Role of the thioredoxin interacting protein in diabetic nephropathy and the mechanism of regulating NOD‑like receptor protein 3 inflammatory corpuscle. Int J Mol Med. 2019;43(6):2440–2450. 31017263 10.3892/ijmm.2019.4163 PMC6488169

[126] Wu M, Yang Z, Zhang C, et al. Inhibition of NLRP3 inflammasome ameliorates podocyte damage by suppressing lipid accumulation in diabetic nephropathy. Metabolism. 2021;118:154748. doi:10.1016/j.metabol.2021.154748. 33675822

[127] Lei F. Optineurin inhibits NLRP3 inflammasome activation by enhancing mitophagy of renal tubular cells in diabetic nephropathy. FASEB J. 2018;33(3):4571–4585. 30571313 10.1096/fj.201801749RRR

[128] Shen J, Dai Z, Li Y, et al. TLR9 regulates NLRP3 inflammasome activation via the NF-κB signaling pathway in diabetic nephropathy. Diabetol Metab Syndr. 2022;14(1):26. doi:10.1186/s13098-021-00780-y. 35120573 PMC8815223

[129] Zhang K, Fan C, Cai D, et al. Contribution of TGF-Beta-Mediated NLRP3-HMGB1 Activation to Tubulointerstitial Fibrosis in Rat With Angiotensin II-Induced Chronic Kidney Disease. Front Cell Dev Biol. 2020;8:1. doi:10.3389/fcell.2020.00001. 32117956 PMC7012792

[130] Krishnan SM, Ling YH, Huuskes BM, et al. Pharmacological inhibition of the NLRP3 inflammasome reduces blood pressure, renal damage, and dysfunction in salt-sensitive hypertension. Cardiovasc Res. 2019;115(4):776–787. doi:10.1093/cvr/cvy252. 30357309 PMC6432065

[131] Bai X, Wang Y, Liu P, et al. Sodium butyrate regulation of NLRP3-Ser295 phosphorylation inhibits hypertensive nephropathy. J Funct Foods. 2023;107:105670. doi:10.1016/j.jff.2023.105670.

[132] Tsai Y-L, Hua K-F, Chen A, et al. NLRP3 inflammasome: pathogenic role and potential therapeutic target for IgA nephropathy. Sci Rep. 2017;7(1):41123. doi:10.1038/srep41123. 28117341 PMC5259731

[133] Hua K-F, Yang S-M, Kao T-Y, et al. Osthole mitigates progressive iga nephropathy by inhibiting ROS generation and NF-κB/NLRP3 pathway. PLoS One. 2013;8(10):e77794. doi:10.1371/journal.pone.0077794. 24204969 PMC3810132

[134] Chun J, Chung H, Wang X, et al. NLRP3 localizes to the tubular epithelium in human kidney and correlates with outcome in IgA nephropathy. Sci Rep. 2016;6(1):24667. doi:10.1038/srep24667. 27093923 PMC4837396

[135] Peng W, Pei G-Q, Tang Y, et al. IgA1 deposition may induce NLRP3 expression and macrophage transdifferentiation of podocyte in IgA nephropathy. J Transl Med. 2019;17(1):406. doi:10.1186/s12967-019-02157-2. 31796125 PMC6891954

[136] Wu C-Y, Hua K-F, Yang S-R, et al. Tris DBA ameliorates IgA nephropathy by blunting the activating signal of NLRP3 inflammasome through SIRT1- and SIRT3-mediated autophagy induction. J Cell Mol Med. 2020;24(23):13609–13622. doi:10.1111/jcmm.15663. 33135320 PMC7753881

[137] Tan J, Dong L, Jiang Z, et al. Probiotics ameliorate IgA nephropathy by improving gut dysbiosis and blunting NLRP3 signaling. J Transl Med. 2022;20(1):382. doi:10.1186/s12967-022-03585-3. 36038927 PMC9422169

[138] Zheng L, Zhu Y, Ma Y, et al. Relationship between hyperuricemia and the risk of cardiovascular events and chronic kidney disease in both the general population and hypertensive patients: a systematic review and meta-analysis. Int J Cardiol. 2024;399:131779. doi:10.1016/j.ijcard.2024.131779. 38218247

[139] Braga TT, Forni MF, Correa-Costa M, et al. Soluble uric acid activates the NLRP3 inflammasome. Sci Rep. 2017;7(1):39884. doi:10.1038/srep39884. 28084303 PMC5233987

[140] Hu Y, Shi Y, Chen H, et al. Blockade of autophagy prevents the progression of hyperuricemic nephropathy through inhibiting NLRP3 inflammasome-mediated pyroptosis. Front Immunol. 2022;13:858494. doi:10.3389/fimmu.2022.858494. 35309342 PMC8924517

[141] Wu Y, He F, Li Y, et al. Effects of shizhifang on NLRP3 inflammasome activation and renal tubular injury in hyperuricemic rats. Evid Based Complement Alternat Med. 2017;2017(1):7674240. doi:10.1155/2017/7674240. 29358971 PMC5735790

[142] Lin X, Zou X, Hu B, et al. Bi Xie Fen Qing Yin decoction alleviates potassium oxonate and adenine induced-hyperuricemic nephropathy in mice by modulating gut microbiota and intestinal metabolites. Biomed Pharmacother. 2024;170:116022. doi:10.1016/j.biopha.2023.116022. 38147734

[143] Hsu CY, McCulloch CE, Iribarren C, et al. Body mass index and risk for ESRD. Ann Intern Med. 2006;144(1):21–28. doi:10.7326/0003-4819-144-1-200601030-00006. 16389251

[144] Boini KM, Xia M, Abais JM, et al. Activation of inflammasomes in podocyte injury of mice on the high fat diet: effects of ASC gene deletion and silencing. Biochim Biophys Acta. 2014;1843(5):836–845. doi:10.1016/j.bbamcr.2014.01.033. 24508291 PMC3986924

[145] Hou X-X, Dong H-R, Sun L-J, et al. Purinergic 2X7 receptor is involved in the podocyte damage of obesity-related glomerulopathy via activating nucleotide-binding and oligomerization domain-like receptor protein 3 inflammasome. Chin Med J (Engl). 2018;131(22):2713–2725. doi:10.4103/0366-6999.245270. 30425198 PMC6247604

[146] Xu X, Huang X, Zhang L, et al. Adiponectin protects obesity-related glomerulopathy by inhibiting ROS/NF-κB/NLRP3 inflammasome pathway. BMC Nephrol. 2021;22(1):218. doi:10.1186/s12882-021-02391-1. 34107901 PMC8191043

[147] Martínez-Klimova E, Aparicio-Trejo OE, Tapia E, et al. Unilateral ureteral obstruction as a model to investigate fibrosis-attenuating treatments. Biomolecules. 2019;9(4):141. doi:10.3390/biom9040141. 30965656 PMC6523883

[148] Tung-Wei H, Yi-Hsien H, Hsiang-Lin L, et al. Renoprotective effect of rosmarinic acid by inhibition of indoxyl sulfate-induced renal interstitial fibrosis via the NLRP3 inflammasome signaling. Int Immunopharmacol. 2024;135:112314. doi:10.1016/j.intimp.2024.112314. 38788450

[149] Honglei G, Ping XB, Shijian Z, et al. NLRP3 deficiency attenuates renal fibrosis and ameliorates mitochondrial dysfunction in a mouse unilateral ureteral obstruction model of chronic kidney disease. Mediators Inflamm. 2017;2017:8316560. doi:10.1155/2017/8316560. PMC5350413 28348462

[150] Vilaysane A, Chun J, Seamone ME, et al. The NLRP3 inflammasome promotes renal inflammation and contributes to CKD. J Am Soc Nephrol. 2010;21(10):1732–1744. doi:10.1681/ASN.2010020143. 20688930 PMC3013544

[151] Liu N, Gao Y, Liu Y, et al. GBP5 inhibition ameliorates the progression of lupus nephritis by suppressing NLRP3 inflammasome activation. Immunol Invest. 2023;52(1):52–66. doi:10.1080/08820139.2022.2122834. 36175170

[152] Yang S-R, Hsu W-H, Wu C-Y, et al. A. Accelerated, severe lupus nephritis benefits from treatment with honokiol by immunoregulation and differentially regulating NF-κB/NLRP3 inflammasome and sirtuin 1/autophagy axis. Faseb J. 2020;34(10):13284–13299. doi:10.1096/fj.202001326R. 32813287

[153] Simona G. NLRP3 inflammasome activation in dialyzed chronic kidney disease patients. PLoS One. 2015;10(3):e0122272–e0122272. 25798846 10.1371/journal.pone.0122272 PMC4370586

[154] Chen Z, Liu L, Gao C, et al. Astragali radix (Huangqi): a promising edible immunomodulatory herbal medicine. J Ethnopharmacol. 2020;258:112895. doi:10.1016/j.jep.2020.112895. 32330511

[155] Sun H, Wang W, Han P, et al. Astragaloside IV ameliorates renal injury in *db/db* mice. Sci Rep. 2016;6(1):32545. doi:10.1038/srep32545. 27585918 PMC5009300

[156] Yuan Y, Yufan W, Minhui H, et al. Astragaloside IV protects against podocyte injury by upregulating mitophagy via the Mfn2/Pink1/parkin axis. Curr Mol Med. 2024;25(7):871–881. doi:10.2174/0115665240310818240531080353. 38867537

[157] Chen Q, Su Y, Ju Y, et al. Astragalosides IV protected the renal tubular epithelial cells from free fatty acids-induced injury by reducing oxidative stress and apoptosis. Biomed Pharmacother. 2018;108:679–686. doi:10.1016/j.biopha.2018.09.049. 30245468

[158] Li X, Dong X, Zhang L, et al. Astragaloside IV attenuates renal tubule injury in DKD rats via suppression of CD36-mediated NLRP3 inflammasome activation. Front Pharmacol. 2024;15:1285797. doi:10.3389/fphar.2024.1285797. 38572426 PMC10987761

[159] Xie J, Ma X, Zheng Y, et al. *Panax notoginseng saponins* alleviate damage to the intestinal barrier and regulate levels of intestinal microbes in a rat model of chronic kidney disease. Ren Fail. 2022;44(1):1948–1960. doi:10.1080/0886022X.2022.2143378. 36354128 PMC9662016

[160] Jing X, Xin M, Xueying L, et al. *Panax notoginseng* Saponins alleviate LPS-induced Fibrosis of HK-2 cells by inhibiting the activation of NLRP3 inflammasome and pyroptosis. Curr Pharm Biotechnol. 2023;25(1):113–123. doi:10.2174/1389201024666230417084507. 37073148

[161] Rauf A, Olatunde A, Imran M, et al. Honokiol: A review of its pharmacological potential and therapeutic insights. Phytomedicine. 2021;90:153647. doi:10.1016/j.phymed.2021.153647. 34362632

[162] Chen Y-Q, Chen H-Y, Tang Q-Q, et al. Protective effect of quercetin on kidney diseases: from chemistry to herbal medicines. Front Pharmacol. 2022;13:968226. doi:10.3389/fphar.2022.968226. 36120321 PMC9478191

[163] Quan Y, Park W, Jin J, et al. Sirtuin 3 activation by honokiol decreases unilateral ureteral obstruction-induced renal inflammation and fibrosis via regulation of mitochondrial dynamics and the renal NF-κB-TGF-β1/Smad signaling pathway. Int J Mol Sci. 2020;21(2):402. doi:10.3390/ijms21020402. 31936371 PMC7014106

[164] Ma Q, Xu M, Jing X, et al. Honokiol suppresses the aberrant interactions between renal resident macrophages and tubular epithelial cells in lupus nephritis through the NLRP3/IL-33/ST2 axis. Cell Death Dis. 2023;14(3):174. doi:10.1038/s41419-023-05680-9. 36859530 PMC9977833

[165] Remsberg CM, Yáñez JA, Ohgami Y, et al. Pharmacometrics of pterostilbene: preclinical pharmacokinetics and metabolism, anticancer, antiinflammatory, antioxidant and analgesic activity. Phytother Res. 2008;22(2):169–179. doi:10.1002/ptr.2277. 17726731

[166] Gu T-T, Chen T-Y, Yang Y-Z, et al. Pterostilbene alleviates fructose-induced renal fibrosis by suppressing TGF-β1/TGF-β type I receptor/Smads signaling in proximal tubular epithelial cells. Eur J Pharmacol. 2019;842:70–78. doi:10.1016/j.ejphar.2018.10.008. 30336139

[167] Wang W, Li K, Bai D, et al. Pterostilbene: a potential therapeutic agent for fibrotic diseases. Inflammopharmacology. 2024;32(2):975–989. doi:10.1007/s10787-024-01440-z. 38429613

[168] Wei W, Bu-Hui L, Yi-Gang W, et al. [Triptolide inhibits NLRP3 inflammasome activation and ameliorates podocyte epithelial-mesenchymal transition induced by high glucose]. Zhongguo Zhong Yao Za Zhi. 2019;44(24):5457–5464. doi:10.19540/j.cnki.cjcmm.20191114.401. 32237395

[169] Chenlei L, Tianyang C, Bingbing Z, et al. Triptolide protects against podocyte injury in diabetic nephropathy by activating the Nrf2/HO-1 pathway and inhibiting the NLRP3 inflammasome pathway. Renal Failure. 2023;45(1):2165103. doi:10.1080/0886022X.2023.2165103. PMC10035962 36938748

[170] Ma CH, Kang LL, Ren HM, et al. Simiao pill ameliorates renal glomerular injury via increasing Sirt1 expression and suppressing NF-κB/NLRP3 inflammasome activation in high fructose-fed rats. J Ethnopharmacol. 2015;172:108–117. doi:10.1016/j.jep.2015.06.015. 26117533

[171] Shui G, Cai Z, Wang F, et al. Simiao pill inhibits epithelial mesenchymal transition in a mouse model of chronic hyperuricemic nephropathy by inhibiting NLRP3 inflammasome activation. BMC Complement Med Ther. 2022;22(1):278. doi:10.1186/s12906-022-03757-0. 36271349 PMC9587568

[172] Lu J-R, Han H-Y, Chen J, et al. Protective effects of bu-shen-huo-xue formula against 5/6 nephrectomy-induced chronic renal failure in rats. Evid Based Complement Alternat Med. 2014;2014:589846. doi:10.1155/2014/589846. PMC4020566 24864155

[174] Østergaard JA, Jha JC, Sharma A, et al. Adverse renal effects of NLRP3 inflammasome inhibition by MCC950 in an interventional model of diabetic kidney disease. Clin Sci (Lond). 2022;136(2):167–180. doi:10.1042/CS20210865. 35048962 PMC8777085

[175] Hsu Y-H, Zheng C-M, Chou C-L, et al. Therapeutic effect of endothelin-converting enzyme inhibitor on chronic kidney disease through the inhibition of endoplasmic reticulum stress and the NLRP3 inflammasome. Biomedicines. 2021;9(4):398. doi:10.3390/biomedicines9040398. 33917140 PMC8067871

